# Analysis of Volatile Aroma Components in Different Parts of Shiitake Mushroom (*Lentinus edodes*) Treated with Ultraviolet C Light-Emitting Diodes Based on Gas Chromatography–Ion Mobility Spectroscopy

**DOI:** 10.3390/molecules29081872

**Published:** 2024-04-19

**Authors:** Daihua Hu, Yulin Wang, Fanshu Kong, Danni Wang, Chingyuan Hu, Xu Yang, Xiaohua Chen, Wang Chen, Zili Feng

**Affiliations:** 1 Vitamin D Research Institute, College of Bioscience and Bioengineering, Shaanxi University of Technology, Hanzhong 723000, China; wangyulin1999@163.com (Y.W.); 19860917312@163.com (F.K.); wdnini@163.com (D.W.); 13571313024@163.com (X.Y.); chenxiaohua@snut.edu.cn (X.C.); 2 Qinba State Key Laboratory of Biological Resources and Ecological Environment, Hanzhong 723000, China; 3 Shaanxi Province Key Laboratory of Bio-Resources, Hanzhong 723000, China; cyhu@snut.edu.cn (C.H.); chenwang@snut.edu.cn (W.C.); 4 Shaanxi Engineering and Technology Research Center for Industrialization of Natural Active Products, Hanzhong 723000, China; fengzili2008@163.com

**Keywords:** shiitake mushrooms, UVC-LED, GC-IMS, volatile compounds, sensory evaluation

## Abstract

Further assessment of ultraviolet C light-emitting diode (UVC-LED) irradiation for influencing shiitake mushrooms’ (*Lentinus edodes*) volatile and sensory properties is needed. In this study, a comparison of UVC-LED irradiation treatment on the flavor profiles in various parts of shiitake mushrooms was conducted using gas chromatography–ion mobility spectrometry (GC-IMS) and sensory analysis. Sixty-three volatile compounds were identified in shiitake mushrooms. The fresh shiitake mushrooms were characterized by the highest values of raw mushroom odors. After UVC-LED treatment, the content of C8 alcohols decreased, especially that of 1-octen-3-ol, while the content of aldehydes increased, especially the content of nonanal and decanal. The score of fatty and green odors was enhanced. For fresh samples, the mushroom odors decreased and the mushroom-like odors weakened more sharply when treated in ethanol suspension than when treated with direct irradiation. The fruit odors were enhanced using direct UVC-LED irradiation for fresh mushroom samples and the onion flavor decreased. As for shiitake mushroom powder in ethanol suspension treated with UVC-LED, the sweaty and almond odor scores decreased and the vitamin D_2_ content in mushroom caps and stems reached 668.79 μg/g (dw) and 399.45 μg/g (dw), respectively. The results obtained from this study demonstrate that UVC-LED treatment produced rich-flavored, quality mushroom products.

## 1. Introduction

Shiitake mushroom (*Lentinus edodes*), a highly nutritious and healthy food, is the world’s second most popular and third most widely cultivated mushroom [1,2]. Shiitake mushrooms are highly favored by consumers worldwide and are widely cultivated in East Asia [3]. Shiitake mushrooms have attracted growing attention in recent years, owing to its satisfying flavor, nutritional features, functional characteristics, and medicinal properties [4,5].

We previously reported on the UV irradiation of shiitake mushroom powder in ethanol suspension. UVC irradiation was more effective than that of UVA and UVB [6,7]. Most conventional UV irradiation sources use mercury lamps to produce shorter wavelength ultraviolet irradiation in traditional vitamin D_2_-enhanced mushroom production [6]. However, mercury is harmful to humans and is easily absorbed into human skin, as well as the respiratory tract and digestive tract of organisms. As a result, the United Nations Minamata Convention formally declared a total ban on manufacturing products containing mercury after 2020 [8]. Thus, searching for new devices to replace mercury lamps as ultraviolet light sources is essential. Ultraviolet C light-emitting diodes (UVC-LED) are the most highly anticipated devices. UVC-LED applications have been used in sterilization [9], water purification [10], air purification [11], and medical fields [12].

Both taste and aroma should be considered when analyzing the overall product flavor for mushrooms. Since volatile compounds contribute to flavor formation, more attention is paid to analyzing volatile compounds in the processing of shiitake mushrooms. However, related research mainly focuses on the effects of different drying methods on volatile compounds. UV-treated mushrooms are available for consumption, but studies exploring the effect of UV treatment on the volatile and sensory properties of treated mushrooms are rare. For example, pulsed light (PL) treatment significantly affected the contents of volatile compounds in shiitake mushrooms [13]. However, a comprehensive analysis coupled with volatile compounds and sensory analysis is missing. Hence, further assessment of UVC-LED irradiation for influencing shiitake mushrooms’ volatile and sensory properties is needed. 

Gas chromatography–ion mobility spectrometry (GC-IMS) has recently been used as a highly sensitive analytical technology for separating and identifying volatile compounds [14,15,16,17]. It has the advantages of easy operation, fast analysis speed, simple sample preparation, and retaining the flavor of samples to the maximum extent [15,17]. In addition, GC-IMS enables an intuitive comparison between samples by establishing a visual fingerprint of volatile compounds and has been widely used in food quality control [18,19], grain and oil storage [20,21], medical diagnosis, and other fields [22,23].

In this study, the comparison of UVC-LED irradiation treatment on flavor profiles in different parts of shiitake mushrooms was conducted using GC-IMS and sensory analysis. The objectives of this work were to (1) establish and characterize the flavor fingerprints of fresh shiitake mushroom caps and stems; (2) investigate the effect of UVC-LED irradiation treatment on the aroma compounds and taste properties of shiitake mushroom caps and stems; (3) establish the differences among the volatile compounds between fresh shiitake mushrooms and mushroom powder in ethanol suspension treated with UVC-LED; and (4) identify the differences among the volatile compounds between UVC-LED treatment at different times for shiitake mushroom caps and stems. The information obtained will be valuable for the flavor research of fresh and dried powder shiitake mushrooms, especially for developing vitamin D_2_-enriched edible fungus products.

## 2. Results and Discussion

### 2.1. GC-IMS Topographic Plots for Various Parts of Shiitake Mushroom Samples Treated Using UVC-LED

The volatile compounds in various parts of shiitake mushroom samples treated using UVC-LED were analyzed using GC-IMS. The data are presented in 3D topographical visualizations (Figure S1), where the *X*-axis is the ion migration time for identification, the *Y*-axis is the t_R_ of the gas chromatograph, and the *Z*-axis is the peak height for quantification. As shown in Figure S1, the volatile substances in various parts of fresh shiitake mushrooms are very similar, but the signal intensity varies. After being treated with UVC-LED, there was little change in volatile compounds for fresh mushrooms. However, most volatile compounds were substantially lost, especially for mushroom powder in ethanol suspension treated with UVC-LED.

**Figure 1:**
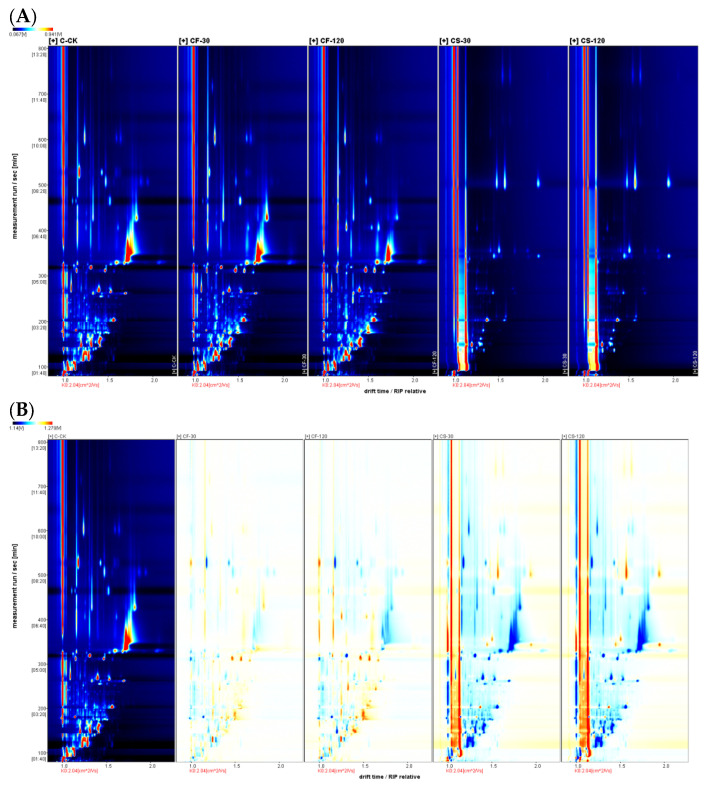
Topographic plots of GC-IMS spectra for shiitake mushroom caps treated with UVC-LED. (**A**) Vertical view; (**B**) comparison view.

**Figure 2:**
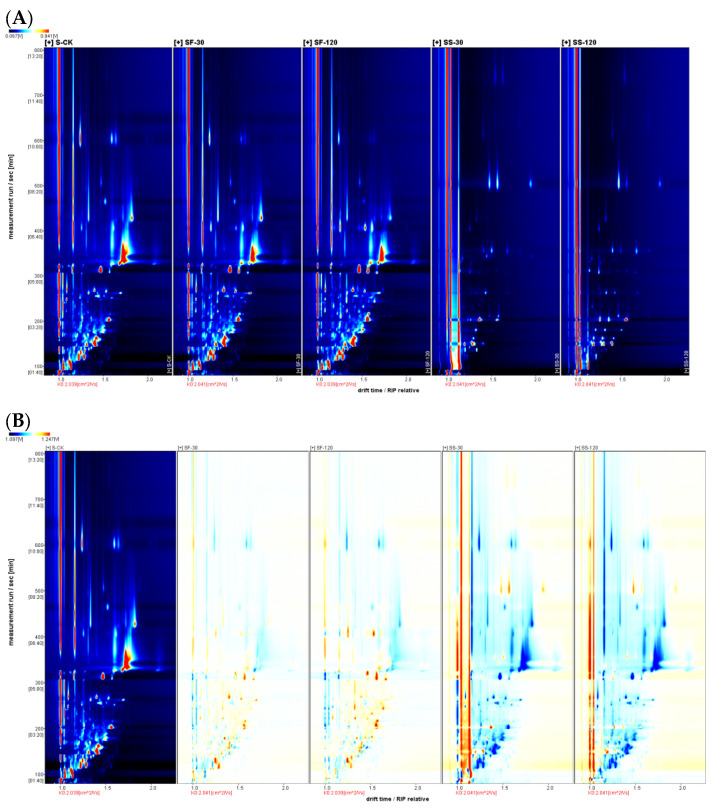
Topographic plots of GC_IMS spectra for shiitake mushroom stems treated with UVC-LED. (**A**) Vertical view; (**B**) comparison view.

The top view of the GC-IMS 3D topographic plots for various parts of shiitake mushroom samples treated with UVC-LED are shown in Figure 1 and Figure 2. The whole spectrum represents the total volatile substances in the mushroom samples. In Figure 1A and Figure 2A, the color in the spectra indicates the signal intensity of the compounds. White represents lower intensity and red indicates higher intensity. The intensity increases as the color deepens.

The difference comparison model further compared the volatile compounds among shiitake mushroom samples. As shown in Figure 1B and Figure 2B, the untreated fresh mushroom caps or stems were selected as a reference in the topographic plot, while the other topographic plot of UVC-LED-treated samples was subtracted from the reference. The white background after deduction indicates that the volatile compounds were consistent. In contrast, a red background indicates a higher concentration of the volatile compound than the background and a blue background indicates a lower concentration of the volatile substance than the control. Figure 1B and Figure 2B show that the differences in volatile compounds in various treatment samples are mainly reflected in the number, concentration, and time of signal peaks. Different mushroom parts and treatment methods resulted in different volatile compounds to a certain degree, similar to the result of *Tricholoma matsutake* [24].

### 2.2. Differences between Volatile Compounds in Various Parts of Shiitake Mushroom Samples Treated with UVC-LED

**Figure 3:**
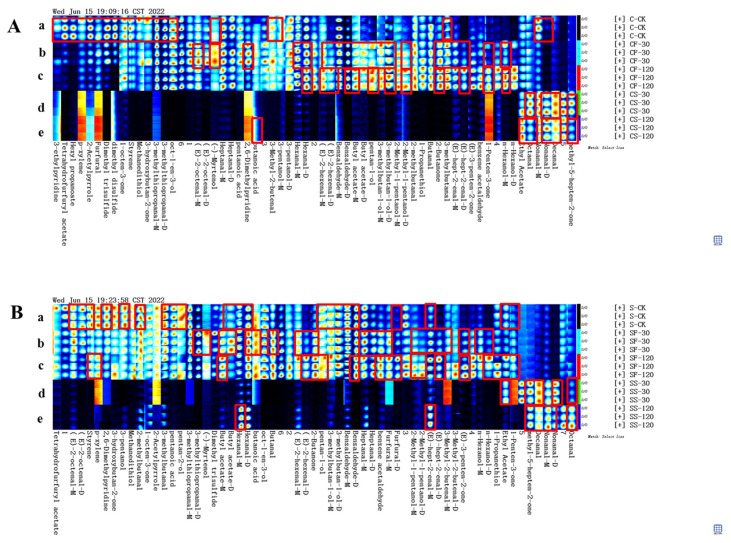
Fingerprint of volatile compounds in various parts of shiitake mushroom treated with UVC-LED. (**A**) Caps samples; (**B**) stem samples. The red frame represents the typical differential volatile compounds, and the lowercase letters a–e represent the corresponding regions of the differential volatile compounds in each samples.

The GC-IMS characterization of volatile compounds in fresh shiitake mushroom caps is presented in Figure S2. Figure 3 shows the fingerprint of volatile compounds in various parts of shiitake mushrooms treated with UVC-LED. Sixty-three volatile substances (monomers and dimers) were identified in shiitake mushroom samples and the corresponding compounds are listed in Table S1. These compounds were classified into six chemical classes, including 28 aldehydes, 13 alcohols, 7 ketones, 5 esters, 2 ethers, 2 acids, 2 pyridines, 1 pyrrole, 1 alkane, 1 alkene, and 1 aromatic compound. The chemical formula of the identified monomer and dimer was the same as the CAS number, but the morphology was different. As shown in Figure 3, UVC-LED treatments altered the volatile profiles of shiitake mushrooms, especially for mushroom powder in ethanol suspension treated with UVC-LED. Table 1 shows the peak volume of forty-one typical volatile compounds identified from shiitake mushrooms with different treatments. Table S2 shows the peak volume of whole volatile compounds identified from shiitake mushrooms with different treatments. Table 2 displays the relative contents of volatile compounds in shiitake mushrooms. After UVC-LED treatment, the contents of various volatile compounds differed significantly (*p* < 0.05), particularly for aldehydes, alcohols, and ethers. Among them, the relative content of aldehydes is 44.75–79.17%, the relative content of alcohols is 4.31–29.40%, the relative content of ketones is 3.71–8.70%, the relative content of esters is 1.63–8.04%, and the relative content of ethers is 0.24–12.61%. After UVC-LED treatment, aldehydes and alcohols were the main volatile compounds in various parts of shiitake mushrooms, followed by ketones, esters, ethers, and other types.

As seen from Table 2, alcohols and aldehydes are the major chemical classes of volatile compounds. Alcohols are mainly derived from amino acid metabolism and the oxidation of unsaturated fats, which have high odor thresholds. An eight-carbon alcohol, such as 1-octen-3-ol, was identified as the main attributor for the typical mushroom-like odors [25]. As shown in Table 1, the content of 1-octen-3-ol varied in different parts of fresh mushrooms. The content of 1-octen-3-ol in the untreated mushroom caps was 12 times higher than in the mushroom stems. UVC-LED treatment has a significant effect on the content of 1-octen-3-ol. After UVC-LED treatment, 1-octen-3-ol in fresh mushroom caps was reduced by 10 times and there was no significant change with the extension of treatment time. Nevertheless, the content of 1-octen-3-ol in mushroom stems had no significant change. In contrast, for mushroom powder, after treatment with UVC-LED in ethanol suspension, the content of 1-octen-3-ol was significantly reduced, by 1360 times.

Aldehydes are the main products of amino acid Strecker degradation or lipid oxidation. Due to their intense odor properties and low odor thresholds, aldehydes also contribute significantly to the characteristic aromas of mushrooms [26]. Eleven kinds of aldehydes, including (E)-2-octenal and benzaldehyde, were significantly decreased with UVC-LED treatment. Among these, the CS-120 samples appeared to have the lowest level of butanal, 113 times lower than in the S-CK samples. 3-methylbutanal has been shown to originate from the Strecker degradation of leucine at high temperatures [27]. Here, 3-methylbutanal appeared with the largest amounts in untreated fresh mushroom stems and the lowest in CS-120 samples. In contrast, the levels of nonanal, decanal, and octanal were increased (*p* < 0.05) with UVC-LED treatment in ethanol suspension. Nonanal is a novel compound, with a fat aroma produced via oleic acid oxidation and drying [28], and it was present in the highest content in CS-120 samples, which was 13 times higher than in the S-CK samples. 

Compared with fresh mushroom stems, the contents of alcohols, esters, and ethers in fresh mushroom caps were higher (*p* < 0.05). In comparison, the contents of aldehydes and other compounds were lower (*p* < 0.05), but the contents of ketones were the same (*p* > 0.05). The volatile compounds in the red region of part a in Figure 3A in fresh mushroom caps were higher than those in fresh mushroom stems (*p* < 0.05). In contrast, the volatile compounds in the red region of part a in Figure 3B in fresh mushroom stems were higher than in fresh mushroom caps (*p* < 0.05).

We analyzed the changes in volatile compounds in various parts of fresh shiitake mushrooms after UVC-LED treatment. The quantities and types of volatile compounds in CF samples were higher for the mushroom caps than in the untreated fresh shiitake mushrooms. UVC-LED treatment elevated the contents of twenty-one volatile compounds (compounds listed in the red region of part b in Figure 3A). Among these compounds, twelve increased (*p* < 0.05) with the extension of UVC-LED treatment time (compounds listed in the red region of part c in Figure 3A). In contrast, (-)-myrtenol and 2,6-dimethylpyridine increased first and then decreased with the extension of processing time (*p* < 0.05). 

The contents of twenty-two volatile compounds (compounds listed in the red region of part b in Figure 3B) increased for the mushroom stems, while eighteen volatile compounds (compounds listed in the red region of part c in Figure 3B) increased (*p* < 0.05) with longer UVC-LED treatment time. 

As shown in Figure 3 and Table 1 and Table 2, the volatile compounds in various parts of shiitake mushroom powder treated with UVC-LED in ethanol suspension were significantly affected (*p* < 0.05). The relative content of alcohols decreased, while the relative content of aldehydes and ethers increased. Compared with the untreated fresh mushrooms, four kinds of volatile compounds in the CS-30 samples (compounds listed in the red region of part d in Figure 3A) of the mushroom caps were increased (*p* < 0.05) following UVC-LED treatment in ethanol suspension. In contrast, six kinds of volatile compounds in the CS-120 samples (compounds listed in the red region of part e in Figure 3A) increased (*p* < 0.05) with longer UVC-LED treatment time. Compared with the untreated fresh mushrooms, five kinds of volatile compounds in the SS-30 samples (compounds listed in the red region of part d in Figure 3B) of the mushroom stems were increased (*p* < 0.05) following UVC-LED treatment in ethanol suspension. In comparison, seven kinds of volatile compounds in the SS-120 samples (compounds listed in the red region of part e in Figure 3B) increased (*p* < 0.05) with longer UVC-LED treatment time.

### 2.3. Similarity Analysis of Volatile Compounds in Various Parts of Shiitake Mushrooms Being Treated with UVC-LED

**Figure 4:**
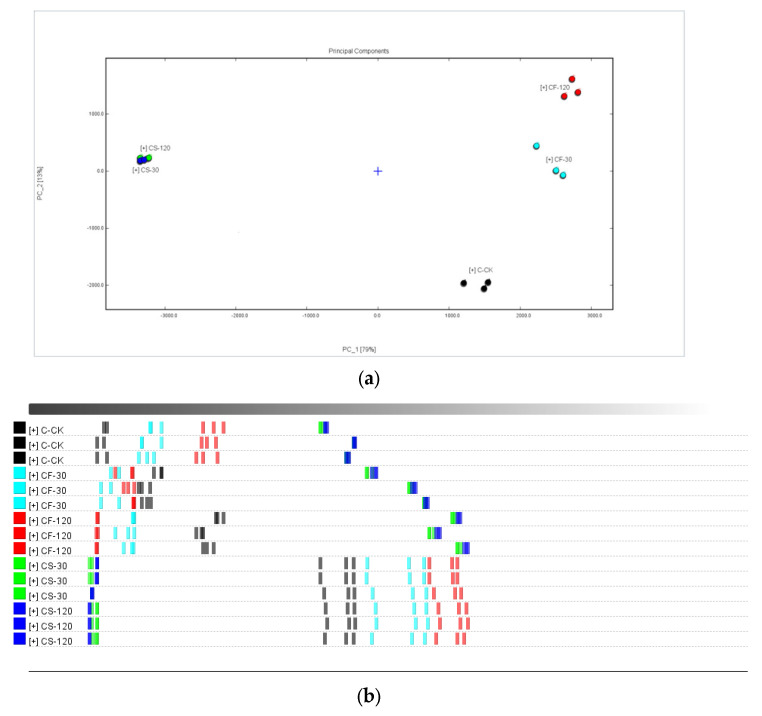
Similarity analysis of volatile compounds in shiitake mushroom caps treated with UVC-LED. (**a**) PCA score plot; (**b**) Euclidean distance diagram.

**Figure 5:**
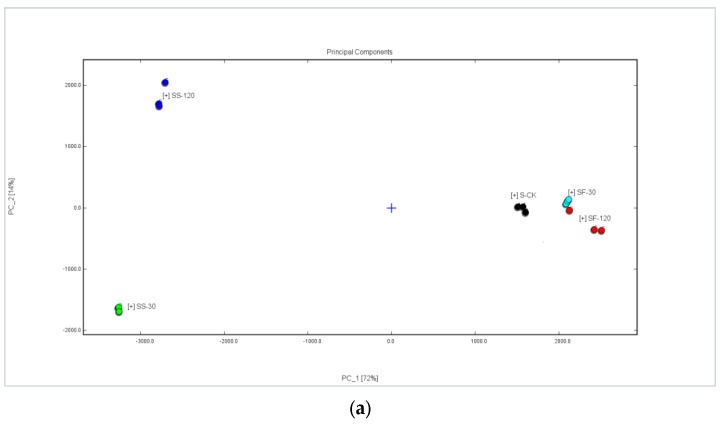

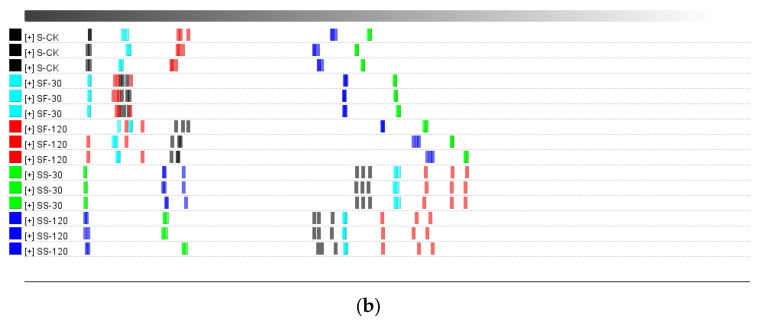
Similarity analysis of volatile compounds in shiitake mushroom stems treated with UVC-LED. (**a**) PCA score plot; (**b**) Euclidean distance diagram.

GC-IMS spectral data, combined with multivariate statistical analysis, have been widely used in the discrimination and identification of agricultural products such as different varieties of sorghum [29] and quinoa with different colors [30,31]. Here, the GC-IMS spectra of volatile composition data in various parts of the shiitake mushrooms being treated with UVC-LED were used for principal component analysis; the results are shown in Figure 4a and Figure 5a.

As shown in Figure 4a, the cumulative contribution of principal components 1 and 2 reached 92% among different treatments, which could represent and explain most of the features of the original data well. The characteristics of volatile compounds in the mushroom samples treated with UVC-LED were different (*p* < 0.05) from those of the untreated mushrooms, while the characteristics of volatile compounds between the CS-30 and CS-120 samples were similar. Nevertheless, the features of volatile compounds varied between the CF-30 and CF-120 samples. Based on the positive score of PC1, the untreated fresh mushroom caps can be defined, and the mushroom caps in ethanol suspension treated with UVC-LED can be identified based on the negative score of PC1. Combined with the PC2 score, we can distinguish the untreated mushroom caps and the samples treated with UVC-LED. Figure 5a shows the cumulative contribution rate of principal component 1 and principal component 2, which reached 86% among different treatments and can better represent and explain most of the features of the original data. There were some differences in volatile substances between the samples treated with UVC-LED and the untreated mushroom stems. The volatile features of the mushroom stems in ethanol suspension treated with UVC-LED were different (*p* < 0.05) from those of the untreated mushroom stems. Among them, the volatile compounds between the SF-30 and SF-120 samples are similar, while the volatile compounds varied between the SS-30 and SS-120 samples. The positive score of PC1 can be used to identify the untreated mushroom stems and the fresh mushroom treated with UVC-LED, while the mushroom stems in ethanol suspension treated with UVC-LED can be identified using the negative score of PC1. Combined with the PC2 scores, we could distinguish the untreated mushroom stems from UVC-LED treatment samples. The mushroom stems in ethanol suspension treated with UVC-LED for different times could also be distinguished.

Figure 4b and Figure 5b are Euclidean distance maps output by the built-in plug-in of the flavor meter. As shown in Figure 4b, the similarity between CF-120 and CS-120 is low at the farthest distance, while the similarity between CS-30 and CS-120 is high at the closest distance. As shown in Figure 5b, the similarity of the distance between SS-30 and SF-120 is low, while the similarity between SF-30 and SF-120 is high. The Euclidean distance of different treatment samples is greater (*p* < 0.05) than the average distance between three duplicate samples. Thus, different treatment samples can be distinguished directly using Euclidean distance.

### 2.4. Sensory Evaluation

**Figure 6:**
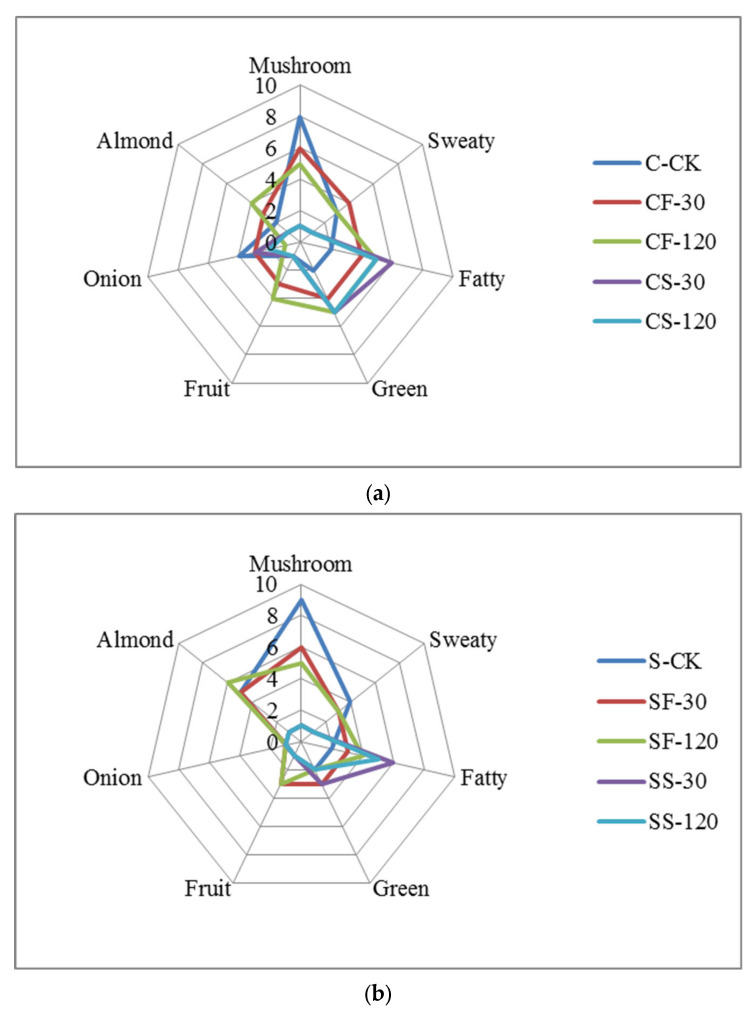
Radar image of seven aroma attributes for shiitake mushrooms subjected to different UVC-LED treatments. (**a**) Shiitake caps samples; (**b**) shiitake stems samples.

The sensory scores of shiitake mushrooms with different UVC-LED treatments are shown in Figure 6. The fresh shiitake was characterized by the highest values of raw mushroom odors, while the odors of mushroom stems were more intense than those of the caps (*p* < 0.05). After UVC-LED treatment, the mushroom odors decreased. Mushroom-like odors weakened more sharply when treated with UVC-LED in ethanol suspension than using direct UVC-LED irradiation for fresh samples. For fresh mushroom caps and stems, the mushroom odors further decreased with the extension of treatment time (*p* < 0.05). For mushroom samples in ethanol suspension treated with UVC-LED, the mushroom-like odors decreased after a short period of treatment (*p* < 0.05), but a longer treatment time did not have any effect (*p* > 0.05). The mushroom flavor properties were positively correlated with the contents of 1-octen-3-ol, 1-octene-3-one, and 3-octanone, etc. [32]. In this study, fresh shiitake mushroom samples were associated with raw mushroom odors, depending on the largest presence of 1-octen-3-ol (70) and 1-octene-3-one (44), which agreed with the published literature on shiitake mushrooms [33].

The sweaty attribute was correlated with acid compounds such as isovaleric acid, hexanoic acid, and heptanal dimer. These compounds have previously been found to be related to sweaty and rancid odor [34]. The sweaty odors elicited by volatile acids were unpleasant and had a negative effect on the overall aroma of shiitake mushrooms [35]. In this study, no evident differences were observed in the sweaty odors score between the control with fresh mushroom stems and caps samples treated with UVC-LED for a short time (30 min). Nevertheless, the sweaty odors score decreased after UVC-LED treatment for a longer time (2 h) (*p* < 0.05). As for shiitake mushroom powder in ethanol suspension treated with UVC-LED, the sweaty odors score decreased after a short time (30 min) (*p* < 0.05). The sweaty odors strengthened in hot air drying (HD) samples and were greatly related to increased contents of volatile acids [33,36]. In this study, the weakening of sweaty odors for UVC-LED treatment samples was consistent with the lowered butanoic and pentanoic acid contents.

The fatty aroma primarily originates from the metabolites of fatty acids, such as the oxidation of fatty acids resulting in the formation of aldehydes that contribute to the development of a fat fragrance. Due to the low odor thresholds and intense odor properties, aldehydes significantly contribute to the characteristic aromas of mushrooms [26]. Five groups of aldehydes, including hexanal, propanal, phenylacetaldehyde, octanal, and propanal dimer, showed the highest contents in fresh samples, due to poor heat processing stability. The signal intensities of nonanal, 3-methylbutanal, and heptanal were increased using three drying methods—HD, VFD, and VFD + HD [33]—consistent with the findings of the published literature on mushrooms [1]. In this current work, the contents of 3-methyl-2-butenal, 2-methylbutanal, E-hept-2-enal, heptanal, hexanal, and E-2-hexenal decreased, while the contents of nonanal and octanal increased after UVC-LED treatment (*p* < 0.05). As a product of oleic acid oxidation, nonanal, imparting a fat aroma, emerged as a novel compound after drying [28]. After UVC-LED treatment, the score of fatty odors was enhanced, which was consistent with the highly increased contents of nonanal, decanal, and octanal in the samples treated with UVC-LED.

The green attribute was correlated with 1-octen-3-ol and ethyl acetate. UVC-LED treatment enhanced (*p* < 0.05) the green flavor of mushrooms. The CF-120, CS-30, and CS-120 samples exhibited higher green properties, consistent with the tendency of content change for 1-octen-3-ol and ethyl acetate among different UVC-LED treatment samples.

The fruit attribute is dependent on the contents of alcohols and ester compounds [33]. The fruit flavor of fresh mushroom caps and stems improved (*p* < 0.05) after UVC-LED treatment. The fruit odor also increased with the extension of treatment time, with the CF-120 sample showing the highest odor. No evident difference in fruit aroma was observed for mushroom samples in ethanol suspension treated with UVC-LED. This result is consistent with the changing trend of the contents of n-hexanol, 3-methylbutan-1-ol, and butyl acetate in different treatment samples.

The almond attribute was correlated with the content of benzaldehyde in shiitake mushrooms. Generally, the shiitake mushroom stems’ almond odor was higher (*p* < 0.05) than that of the caps. No evident difference in almond aroma was observed for fresh mushroom caps with extended UVC-LED treatment time. Nevertheless, the almond odors increased (*p* < 0.05) with extended UVC-LED treatment time for fresh mushroom stems, while the SF-120 sample had a considerably higher (*p* < 0.05) level of almond aroma. The almond odors decreased for shiitake caps and stems in ethanol suspension treated with UVC-LED (*p* < 0.05). This result is consistent with the changing trend of benzaldehyde content in different treatment samples.

The unique onion and slightly sulfurous attributes are related to sulfur-containing compounds in shiitake mushrooms [36]. The main characteristic sulfur-containing compounds in fresh shiitake mushrooms include 1,2,3,5,6-pentamethiocycloheptane (Lenthionine), 1,2,4-trithioheterocyclopentane, dimethyl disulfide (DMDS), and dimethyl trisulfide (DMTS). DMDS and DMTS are typical high-temperature decomposition products of lenthionine [1]. After heat treatment, DMDS and DMTS might also be reduced by rapid evaporation or transformation into cyclic compounds (e.g., 2,6-dimethyl pyrazine, 2,3,5-trimethyl pyrazine, and 2-ethyl furan) [36]. In this study, the onion flavor of fresh mushroom caps was the most intense and was higher than that of mushroom stems (*p* < 0.05). After the UVC-LED treatment, the onion flavor was reduced (*p* < 0.05). The onion flavor for fresh mushrooms decreased with extended UVC-LED treatment time (*p* < 0.05). In contrast, a longer treatment time had no effect (*p* > 0.05) on the onion flavor for shiitake mushrooms in ethanol suspension treated with UVC-LED. This observation is consistent with the changes in the content of DMDS and DMTS in different treatment samples. 

### 2.5. Effect of UVC-LED Treatment on Vitamin D_2_ and Ergosterol Contents in Various Parts of Shiitake Mushrooms

Vitamin D_2_ was undetectable in the unexposed fresh shiitake mushroom caps and stems. The ergosterol content in the caps samples was higher (*p* < 0.05) than that of the stem samples. The ergosterol concentration was as high as has been reported for oyster mushrooms [6] and enoki mushrooms [37].

For the fresh mushroom caps, the concentration of vitamin D_2_ increased (*p* < 0.05) from 26.55 μg/g (dw) to 48.12 μg/g (dw) with extended UVC-LED treatment time. When treated for 30 min, the ergosterol content decreased (*p* < 0.05) and the retention rate of ergosterol was only 50.03%. For the fresh mushroom stems, the vitamin D_2_ content increased (*p* < 0.05) from undetectable to 29.25 μg/g (dw) with UVC-LED treatment, but a longer treatment time did not affect vitamin D_2_ and ergosterol content (Table 3). The ergosterol content in the caps and stems of fresh mushrooms both increased (*p* > 0.05) with extended UVC-LED treatment time. This result is consistent with the changing trend of literature reports [6,7,37].

For the mushroom caps powder, when treated with UVC-LED in ethanol suspension, the vitamin D_2_ content increased (*p* < 0.05), while the concentration of ergosterol decreased (*p* < 0.05), with extended treatment time. After being treated with UVC-LED for 30 min and 120 min, the vitamin D_2_ content reached 267.85 μg/g(dw) and 668.79 μg/g (dw), which shows an approximately 10.08-fold and 13.89-fold increase compared with the CF-30 and CF-120 samples, respectively. The ergosterol retention rates reached 60.70% and 37.51%, respectively (Table 3).

The vitamin D_2_ content increased (*p* < 0.05) when treated with UVC-LED for the mushroom stem powder in ethanol suspension. However, a longer treatment time did not affect the ergosterol concentration (*p* > 0.05). After treating with UVC-LED for 30 min and 120 min, the vitamin D_2_ content reached 112.60 μg/g (dw) and 399.45 μg/g (dw), an approximately 3.85-fold and 16.02-fold increase compared with the SF-30 and SF-120 samples, respectively. The ergosterol retention rates reached 46.41% and 37.73%, respectively (Table 3).

The UVC-LED light is more efficient than the high-pressure mercury lamp for converting ergosterol to vitamin D_2_ in shiitake mushrooms. In our previous study [6], the concentration of vitamin D_2_ in shiitake mushrooms increased to 392.53 μg/g (dw) and 677.28 μg/g (dw) after exposure for 30 min and 120 min in ethanol suspension. In the previous study, the high-pressure mercury lamp was used as the UV light source, with an irradiation intensity of 3.29 mW/cm^2^, and the calculated irradiation doses after 30 min and 120 min were 59.22 kJ/m^2^ and 236.88 kJ/m^2^, respectively. In this study, the LED was used as the UV light source, with an irradiation intensity of 195 μW/cm^2^, and the calculated irradiation doses after 30 min and 120 min were 3.51 kJ/m^2^ and 14.04 kJ/m^2^, respectively. The concentration of vitamin D_2_ in shiitake mushroom caps increased to 267.85 μg/g (dw) and 668.79 μg/g (dw) after exposure for 30 min and 120 min in ethanol suspension. When exposed for 120 min, the UV radiation dose of the high-pressure mercury lamp was 16.87 times that of the LED, but there was no significant difference in vitamin D_2_ content between them.

## 3. Materials and Methods

### 3.1. Materials

**Figure 7:**
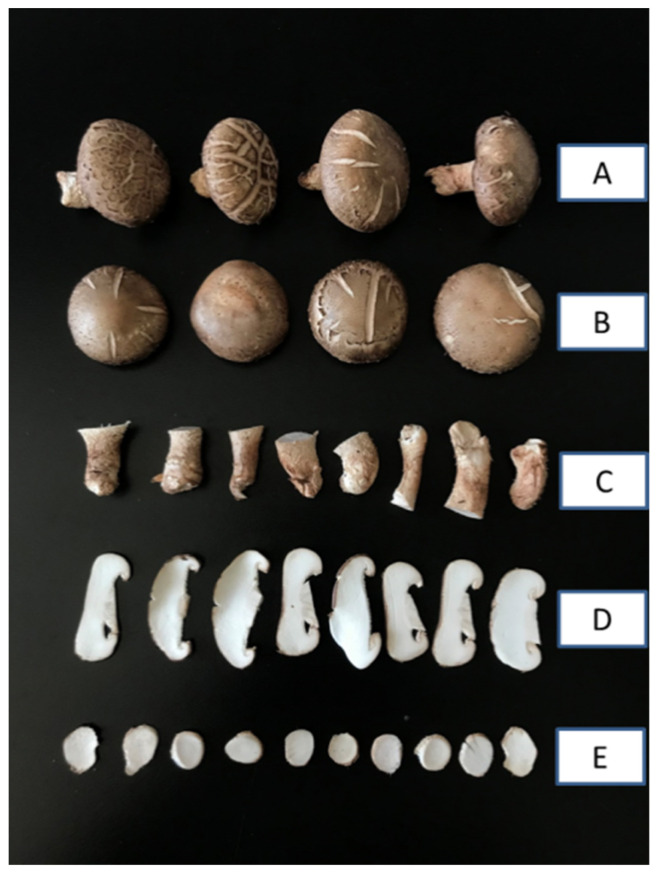
Fresh shiitake mushrooms of different groups. (**A**) Whole shiitake mushrooms; (**B**) unsliced cap; (**C**) unsliced stem; (**D**) sliced cap; and (**E**) sliced stem.

Fresh cultivated shiitake mushrooms with uniform maturity and size were purchased from a local supermarket supplied by Keming Co. (Lueyang, Shaanxi, China, 33.33 N and 106.15 E). After the removal of the surface dirt, fresh shiitake mushroom samples were divided into two parts—caps and stem—and the two parts were cut into 2 mm slices using a slicer (LG-814, Baixin Co., Wenzhou, China), respectively. The different treatment groups of shiitake mushrooms are shown in Figure 7. The fresh shiitake mushroom samples were recorded as caps control (C-CK) and stem control (S-CK).

The moisture content of fresh shiitake mushrooms was determined using the hot air oven method, as described previously [38]. Briefly, mushrooms were dried at 105 °C for 6–8 h using an electric–thermostatic blast oven (101-3A dry oven, Taisite Co., Tianjin, China) until they reached a constant weight. The moisture content of fresh shiitake was 86.94 ± 0.38% (wet basis) for the caps and 81.28 ± 0.20% (wet basis) for the stems, respectively.

### 3.2. Chemicals

N-ketones (C4–C9) (Sinopharm Chemical Reagent Co., Ltd., Beijing, China) were used as external references to calculate the retention index (RI) of volatile compounds. The standard compounds of vitamin D_2_ (99.6%) and ergosterol (95.5%) were purchased from Meilun Reagent Co., Ltd., (Xi’an, China). Chromatographic grade acetonitrile and formic acid were purchased from Aladdin Reagent Co., Ltd., (Shanghai, China). Deionized water was obtained from a molecular ultrapure water system (Molewater System Co., Ltd., Chongqing, China) with an electrical resistivity of 18.25 MΩ·cm.

### 3.3. UVC-LED Irradiation

#### 3.3.1. UVC-LED Irradiation for Fresh Shiitake Mushroom

Freshly sliced shiitake mushroom caps and stems were evenly spread on a stainless steel tray. The sliced mushrooms were exposed to UVC-LED irradiation with an irradiation intensity of 195 μW/cm^2^ (HP350UV Spectral illuminometer, Hangzhou LCE Intelligent Detection Instrument Co., Ltd., Hangzhou, China) at a distance of 30 cm from the UV-LED lamps in an LED Multispectral Ultraviolet experiment chamber (XL-UV001, Tsingtao Jingyuanfanguang Co., Ltd., Tsingtao, China,) for 30 min and 120 min. The UV wavelength was selected as 275 nm. The calculated irradiation dose after 30 min and 120 min exposure periods was 3.51 kJ/m^2^ and 14.04 kJ/m^2^, respectively. Sliced mushrooms were turned every 15 min. The treated cap mushroom samples were designated CF-30 and CF-120, while the stem mushroom treated samples were designated SF-30 and SF-120, respectively. The fresh and dried samples were wrapped in silver paper and stored at −80 °C to prepare for the subsequent analyses.

#### 3.3.2. UVC-LED Irradiation for Shiitake Mushroom Powder in Ethanol Suspension

The dried caps and stems of shiitake mushrooms were ground into a powder (FW177 mill, Taisite Co., Tianjin, China) and were screened using a 60-mesh sieve. A total of 10.0 g of caps (or stems) mushroom powder and anhydrous alcohol were mixed in a 250 mL glass beaker with a material–liquid ratio of 1:20 (g/mL). The mixtures were exposed to UV-LED irradiation with an irradiation intensity of 195 μW/cm^2^ (HP350UV Spectral illuminometer, Hangzhou LCE Intelligent Detection Instrument Co., Ltd., Hangzhou, China) at a distance of 30 cm from the UV-LED lamps in an LED Multispectral Ultraviolet experiment chamber (XL-UV001, Tsingtao Jingyuanfanguang Co., Ltd., Tsingtao, China) for 30 min and 120 min with magnetic stirring at 500 rpm (CMAG HS7 digital magnetic stirrer, IKA Co., Staufen, Germany). The UV wavelength was selected as 275 nm. The calculated irradiation dose after 30 min and 120 min exposure periods was 3.51 kJ/m^2^ and 14.04 kJ/m^2^, respectively. Then, the solvent was removed completely (RV10 Rotary Evaporator, IKA Co., Staufen, Germany). The obtained mushroom cap irradiated solids were named CS-30 and CS-120, while the obtained mushroom stem irradiated solids were named SS-30 and SS-120 [6].

### 3.4. Drying Procedures

The fresh sliced shiitake mushrooms were dried using the freeze drying method, according to the literature [38], but with some modifications. The pre-freezing stage was −45 °C for 6 h and the sublimation stage was −30 °C for 5 h, −20 °C for 3 h, 0 °C for 3 h, and 15 °C for 24 h, respectively, performed using a freeze-dryer (LGJ-40G, Foring Technology Development Co., Ltd., Beijing, China).

### 3.5. GC-IMS Analysis

The determination of volatile compounds in shiitake mushroom samples was completed using a GC-IMS (FlavourSpec, G.A.S., Dortmund, Germany) method [16]. Fresh samples (2.0 g) and finely ground dried samples (0.2 g) were placed into a 20 mL headspace sampling vial. After incubating at 60 °C for 10 min, 500 μL of headspace was automatically injected through a heated syringe (85 °C) in splitless mode. The flow rate program of the carrier gas with 99.999% purity of nitrogen was as follows: 2 mL/min for 2 min, 20 mL/min for 8 min, 50 mL/min for 10 min, and 150 mL/min for 5 min. The analytes were driven to the ionization chamber and ionized using a positive ion mode. Then, the resulting ions were driven to the drift tube (5.0 cm in length). The volatile compounds were identified by comparing the RI (retention index) and Dt (drift time—the time it takes for ions to reach the collector through the drift tube, in milliseconds) time through the GC-IMS library. The RI of each compound was determined using C4–C9 n-ketones. The retention time (t_R_) and Dt were used for qualitative analysis, and the peak volume (which refers to the integration of peak height in the two dimensions of retention time and drift time) was used for quantitative analysis. All analyses were performed in triplicate.

### 3.6. Sensory Evaluation

The sensory group comprises fifteen panelists (nine females and six males, aged 20–40) from the College of Biological Sciences and Engineering, all of whom were trained members of an established sensory panel, who had passed an odor recognition test and were familiar with the descriptors and methodologies. The aromas were classified into seven attributes (mushroom, fatty, fruit, sweaty, green, onion, and almond), according to the reported method [39], with minor modifications. The descriptors were compared with the following reference materials: mushroom-like (1-octen-3-ol), fatty-like (nonanal), fruit (butyl acetate), sweaty (3-methylbutanoic acid), green (ethyl acetate), onion (dimethyl disulfide), and almond (benzaldehyde). With the attribute-by-attribute method, only one reference and all samples were provided to panelists in each session. To minimize the carry-over effect, a 2 min rest period was enforced between samples. The panelists were asked to score the perceived intensities of the selected aroma attributes based on a 10-point scale, where 0 indicates the absence and 10 indicates the highest intensity. All 10 samples were assessed in triplicate by each panelist.

### 3.7. Vitamin D_2_ and Ergosterol Determinations

Vitamin D_2_ and ergosterol were extracted and analyzed using a modified method, as reported previously [6,7]. The standard curve was obtained using series standard solutions of vitamin D_2_ and ergosterol. The quantification of vitamin D_2_ and ergosterol was achieved by extrapolation from a standard curve. The samples of mushroom powder (0.2 g) were ultrasonically extracted with 3 mL of ethanol for 25 min at 50 °C, three times. The ethanol phase was then collected and diluted with ethanol to 10 mL. The extract was filtered through a 0.45 μm filter, before undergoing high-performance liquid chromatography (HPLC) analysis. A volume of 10 μL of filtered sample was directly injected into an HPLC system (1260, Agilent Co., Santa Clara) and was eluted through a reversed-phase C18 column (Zorbax 300 extend-C18, 4.6 × 100 mm (3.5 μm), Agilent Co., Santa Clara, CA, USA). The mobile phase for HPLC was acetonitrile/0.1% formic acid (95:5, *v*/*v*) in a constant program at a flow rate of 1.0 mL/min. The UV detection was set at 264 nm. 

### 3.8. Statistical Analysis

All experiments were conducted in triplicate, with the data expressed as mean value ± standard derivation (SD). The instrumental analysis was performed using GC-IMS Library Search, Reporter Gallery plot, and 2.2.1 LAV (Laboratory Analytical Viewer) processing software (FlavourSpec^®^, G.A.S., Dortmund, Germany), which can be used for sample analysis from different angles. Analysis of variance (ANOVA) was used to analyze the data. Duncan’s HSD was used to determine the significant differences between treatments. Statistical analysis was conducted using SPSS 16.0 for Windows. A significance level of *p* < 0.05 was used to separate the mean for all treatments.

## 4. Conclusions

In this study, the comparison of UVC-LED irradiation treatment on flavor profiles in various parts of shiitake mushrooms was conducted using GC-IMS and sensory analysis. Results show that 63 volatile compounds were identified in shiitake mushrooms. Aldehydes and alcohols were the main types of volatile flavor substances after UVC-LED treatment, followed by ketones, ethers, and esters. After UVC-LED treatment, the contents of C8 alcohols decreased, especially for 1-octen-3-ol, while the contents of aldehydes increased, especially for nonanal and decanal. Principal component and Euclidean distance analysis show that GC-IMS could distinguish volatile substances in various parts of shiitake mushrooms. 

The fresh shiitake was characterized by the highest values of raw mushroom odors, while the odors of mushroom stems were more intense than those of the caps (*p* < 0.05). After UVC-LED treatment, the score of fatty and green odors was enhanced. The mushroom odors decreased. Mushroom-like odors weakened more sharply when treated with UVC-LED in ethanol suspension than using direct irradiation for fresh samples. The fruit odors were enhanced (*p* < 0.05) for fresh mushroom samples and the onion flavor decreased. As for shiitake mushroom powder in ethanol suspension treated with UVC-LED, the sweaty and almond odors score decreased and the vitamin D_2_ content in mushroom caps and stems reached 668.79 μg/g (dw) and 399.45 μg/g (dw), respectively. 

The results obtained from this study demonstrate that UVC-LED treatment produced rich-flavored quality mushroom products. The information obtained is valuable for the flavor research of fresh and dried powder shiitake mushrooms, especially for developing vitamin D_2_-enriched edible fungus products and for the further assessment of UVC-LED irradiation for influencing shiitake mushrooms’ volatile and sensory properties.

## Figures and Tables

**Table 1:** Peak volume of forty-one typical volatile compounds identified from shiitake mushroom with different treatments.

Count	Compound	Peak Volume									
		C-CK	S-CK	CF-30	SF-30	CF-120	SF-120	CS-30	CS-120	SS-30	SS-120
2	Nonanal-M	1121.87 ± 123.36 bc	516.68 ± 24.14 d	560.05 ± 18.70 d	471.88 ± 11.39 d	580.09 ± 8.82 d	470.64 ± 11.70 d	1217.54 ± 24.74 b	990.63 ± 18.69 c	1165.60 ± 35.60 b	1406.95 ± 72.82 a
4	(E)-2-octenal-M	1168.89 ± 82.60 d	1439.12 ± 51.14 abc	1522.94 ± 154.57 ab	1613.89 ± 54.92 a	1370.27 ± 39.28 bc	1267.03 ± 47.78 cd	96.14 ± 8.12 e	91.55 ± 3.36 e	159.89 ± 8.52 e	278.88 ± 16.30 e
5	(E)-2-octenal-D	1697.18 ± 164.73 bc	2554.85 ± 171.59 a	2135.44 ± 388.78 ab	2071.31 ± 111.03 b	1173.13 ± 88.40 d	1411.05 ± 101.22 cd	65.86 ± 18.53 e	54.98 ± 8.05 e	63.37 ± 13.20 e	59.04 ± 9.26 e
7	benzene Acetaldehyde	379.25 ± 93.68 cd	481.88 ± 48.34 cd	586.78 ± 125.12 c	1139.66 ± 59.77 b	1379.87 ± 170.48 a	1607.23 ± 50.99 a	244.10 ± 7.28 d	252.32 ± 3.16 d	251.68 ± 4.45 d	287.97 ± 6.43 d
9	Nonanal-D	272.84 ± 52.40 e	88.75 ± 6.61 f	87.14 ± 5.61 f	81.51 ± 2.46 f	92.97 ± 7.73 f	78.77 ± 6.15 f	1081.37 ± 87.72 b	1241.88 ± 61.88 a	714.00 ± 18.51 c	414.17 ± 33.32 d
11	Decanal	95.36 ± 11.46 d	83.20 ± 3.73d e	75.37 ± 2.82 e	65.86 ± 4.10 e	72.25 ± 1.06 e	66.80 ± 5.30 e	247.95 ± 8.09 a	193.45 ± 7.85 b	191.87 ± 3.24 b	144.32 ± 4.29 c
13	Dimethyl trisulfide	1939.56 ± 210.48 a	75.28 ± 14.95 cd	1317.78 ± 179.27 b	136.24 ± 29.19 cd	320.56 ± 8.50 c	110.19 ± 11.91 cd	19.67 ± 3.54 e	22.55 ± 3.76 e	21.96 ± 4.91 e	23.99 ± 3.43 e
14	Benzaldehyde-M	1181.71 ± 74.24 c	1705.72 ± 21.93 a	1519.95 ± 77.50 b	1551.58 ± 58.27 b	1535.48 ± 64.81 b	1127.15 ± 6.67 c	617.44 ± 34.38 e	885.01 ± 3.00 d	535.16 ± 5.48 e	375.28 ± 15.75 f
15	Benzaldehyde-D	1309.61 ± 201.87 e	5967.98 ± 89.55 b	2769.03 ± 267.23 d	5911.48 ± 252.61 b	3537.58 ± 370.12 c	7826.78 ± 305.84 a	63.54 ± 11.97 f	65.07 ± 8.52 f	86.55 ± 10.32 f	97.19 ± 12.23 f
18	Heptanal-M	538.02 ± 25.97 bc	497.75 ± 6.49 c	584.33 ± 31.76 ab	569.29 ± 12.55 b	629.38 ± 18.00 a	541.30 ± 17.20 bc	83.13 ± 2.44 e	82.05 ± 3.74 e	89.25 ± 2.92 e	255.95 ± 25.39 d
19	Heptanal-D	690.93 ± 76.69 ab	466.91 ± 27.29 b	625.21 ± 158.82 ab	745.46 ± 30.19 a	718.12 ± 76.75 ab	870.98 ± 170.07 a	41.27 ± 8.12 c	66.50 ± 4.50 c	33.41 ± 1.56 c	70.78 ± 6.96 c
21	pentanoic acid	204.76 ± 6.90 b	288.42 ± 2.33 a	233.89 ± 10.74 b	228.16 ± 6.69 b	160.93 ± 1.18 c	207.36 ± 29.44 b	14.24 ± 2.90 d	14.11 ± 3.46 d	13.53 ± 2.37 d	24.58 ± 4.61 d
22	Hexanal-M	618.48 ± 25.93 c	737.40 ± 4.53 b	709.01 ± 17.57 b	702.42 ± 5.88 b	698.98 ± 25.77 b	593.26 ± 24.45 c	228.17 ± 6.00 e	190.49 ± 4.53 e	335.94 ± 0.84 d	978.51 ± 30.60 a
23	Hexanal-D	2452.06 ± 73.18 e	2889.71 ± 74.46 d	3268.57 ± 144.72 c	3971.58 ± 24.78 ab	3709.24 ± 110.91 b	4103.04 ± 178.64 a	366.11 ± 7.92 h	315.11 ± 8.66 h	729.43 ± 30.78 g	2032.37 ± 86.84 f
24	2,6-Dimethylpyridine	64.64 ± 5.38 e	463.89 ± 7.79 a	138.36 ± 2.93 d	308.28 ± 8.41 b	74.94 ± 7.02 e	242.34 ± 4.52 c	16.54 ± 0.57 f	15.58 ± 0.87 f	16.35 ± 0.94 f	7.63 ± 1.86 f
25	Furfural-M	100.72 ± 13.37 c	120.93 ± 14.26 c	101.73 ± 19.38 c	261.37 ± 31.57 b	88.32 ± 6.84 c	321.28 ± 27.19 a	24.37 ± 0.39 d	21.62 ± 0.29 d	23.06 ± 1.10 d	19.52 ± 1.70 d
27	(E)-2-hexenal-M	121.29 ± 11.45 d	152.87 ± 7.28 d	189.95 ± 8.20 c	249.37 ± 1.48 ab	229.84 ± 9.64 b	262.47 ± 25.82 a	35.91 ± 1.64 e	41.70 ± 2.49 e	40.69 ± 1.39 e	128.48 ± 7.16 d
29	Butanoic acid	34.27 ± 8.30 cd	38.68 ± 7.69 cd	47.61 ± 3.26 bc	53.58 ± 1.87 b	40.60 ± 2.31 bcd	45.68 ± 6.32 bc	46.42 ± 2.98 bc	69.20 ± 1.41 a	30.17 ± 0.82 d	7.32 ± 1.04 e
31	(E)-hept-2-enal-M	142.41 ± 13.90 de	178.22 ± 11.24 c	319.63 ± 17.83 a	245.20 ± 5.30 b	351.39 ± 14.15 a	273.04 ± 15.24 b	107.15 ± 2.01 f	113.12 ± 1.94 ef	163.73 ± 1.72 cd	325.56 ± 8.09 a
32	(E)-hept-2-enal-D	46.13 ± 4.42 c	103.62 ± 7.19 c	263.01 ± 28.58 b	323.68 ± 34.64 b	489.49 ± 91.53 a	472.03 ± 73.36 a	19.21 ± 3.75 c	24.69 ± 1.98 c	30.92 ± 4.34 c	68.96 ± 4.97 c
34	n-Hexanol-M	59.00 ± 5.57 c	157.05 ± 1.42 c	383.12 ± 75.98 b	368.33 ± 16.53 b	651.51 ± 21.53 a	489.45 ± 98.70 b	43.48 ± 2.19 c	49.63 ± 1.82 c	43.87 ± 4.58 c	48.40 ± 5.66 c
35	n-Hexanol-D	10.04 ± 1.06 c	15.72 ± 1.20 c	81.98 ± 31.32 bc	95.12 ± 5.49 b	264.23 ± 14.76 a	242.05 ± 61.75 a	7.73 ± 1.05 c	7.76 ± 1.32 c	8.99 ± 1.68 c	7.50 ± 1.64 c
36	Furfural-D	76.61 ± 6.25 de	181.83 ± 4.04 bc	116.26 ± 4.81 cd	249.09 ± 48.42 b	112.30 ± 6.47 cd	445.62 ± 57.60 a	40.67 ± 4.75 de	42.04 ± 3.86 de	39.03 ± 4.67 de	27.73 ± 5.15 e
37	(E)-2-hexenal-D	27.52 ± 3.72 ef	53.66 ± 2.95 de	83.78 ± 10.10 cd	110.00 ± 10.99 bc	127.76 ± 17.71 b	226.93 ± 27.99 a	14.89 ± 2.42 f	11.48 ± 1.96 f	14.08 ± 1.89 f	19.49 ± 2.94 ef
38	Butyl acetate-M	119.52 ± 7.23 d	129.95 ± 1.83 d	170.68 ± 8.33 c	210.92 ± 4.21 b	212.22 ± 8.27 b	242.73 ± 2.18 a	33.71 ± 2.32 f	31.78 ± 1.99 f	35.67 ± 1.46 f	67.77 ± 3.22 e
40	Octanal	60.30 ± 1.75 d	49.91 ± 0.87 d	51.95 ± 9.19 d	67.64 ± 6.67 d	59.32 ± 4.80 d	57.50 ± 16.00 d	163.80 ± 1.95 b	117.29 ± 0.83 c	140.04 ± 3.61 bc	272.04 ± 20.00 a
44	1-Octen-3-one	112.06 ± 5.94a	74.90 ± 2.61d	68.60 ± 7.96d	82.46 ± 3.19cd	103.42 ± 9.89ab	93.74 ± 6.26bc	26.39 ± 1.87e	23.24 ± 2.78e	31.17 ± 0.93 e	75.49 ± 1.79 d
47	Dimethyl disulfide	1212.54 ± 88.71 a	43.57 ± 1.05 e	480.13 ± 29.66 d	52.14 ± 3.87 e	60.25 ± 6.07 e	77.64 ± 6.30 e	1039.04 ± 12.94 b	1165.34 ± 4.57 a	898.34 ± 8.86 c	135.80 ± 11.10 e
50	Pentan-1-ol	170.56 ± 13.90 e	525.63 ± 10.55 c	657.20 ± 63.22 b	424.68 ± 12.36 d	840.45 ± 49.90 a	540.40 ± 8.00 c	18.54 ± 4.42 f	19.06 ± 4.86 f	18.69 ± 4.85 f	23.56 ± 5.97 f
51	3-Methyl-2-butenal-M	60.74 ± 1.88 ab	44.09 ± 0.62 c	47.29 ± 7.97 bc	60.47 ± 0.98 ab	47.13 ± 3.31 bc	62.49 ± 9.68 a	29.54 ± 1.49 d	28.58 ± 1.78 d	30.25 ± 2.04 d	17.65 ± 1.55 d
52	3-Methylbutan-1-ol-D	391.74 ± 79.46 d	591.83 ± 12.98 c	916.28 ± 101.16 b	663.29 ± 2.42 c	1107.61 ± 73.74 a	702.73 ± 26.14 c	20.98 ± 3.89 e	15.13 ± 1.99 e	20.32 ± 1.92 e	17.93 ± 1.33 e
53	Butyl acetate-D	12.01 ± 0.92 d	52.89 ± 2.90 b	34.65 ± 2.17 c	77.43 ± 10.01 a	33.11 ± 4.42 c	38.30 ± 1.98 c	5.22 ± 1.04 d	5.64 ± 1.87 d	7.11 ± 1.12 d	6.87 ± 2.67 d
55	3-Methyl-2-butenal-D	81.43 ± 5.08 ab	52.63 ± 2.15 b	75.13 ± 16.42 ab	76.17 ± 1.85 ab	56.85 ± 6.54 b	102.57 ± 32.82 a	7.43 ± 0.27 c	8.79 ± 0.86 c	9.96 ± 1.82 c	15.85 ± 3.75 c
56	3-Methylbutan-1-ol-M	53.20 ± 4.10 c	88.17 ± 6.12 b	139.59 ± 16.45 a	104.54 ± 0.16 b	132.76 ± 7.38 a	90.52 ± 20.47 b	27.33 ± 1.88 cd	19.89 ± 1.88 d	30.52 ± 3.07 cd	35.52 ± 2.66 cd
59	2-Methylbutanal-D	1720.30 ± 67.75 b	2178.78 ± 48.23 a	1668.37 ± 110.67 b	1871.52 ± 29.66 b	1784.22 ± 87.06 b	1744.87 ± 123.04 b	60.93 ± 7.14 d	51.11 ± 3.11 d	193.35 ± 6.47 d	737.99 ± 61.36 c
61	Butanal	2682.47 ± 518.61 b	2409.04 ± 178.95 b	2081.47 ± 241.56 b	4111.36 ± 53.03 a	3568.64 ± 166.36 a	4146.59 ± 247.11 a	25.59 ± 5.47 c	21.05 ± 4.91 c	29.04 ± 6.09 c	67.72 ± 12.81 c
62	3-Methylbutanal-D	898.04 ± 95.95 d	2306.82 ± 17.47 a	1336.14 ± 128.46 c	1994.18 ± 62.13 b	1594.98 ± 59.03 c	1501.35 ± 211.00 c	36.41 ± 3.38 f	27.54 ± 2.98 f	139.93 ± 3.26 f	552.40 ± 19.94 e
64	Ethyl Acetate	235.71 ± 12.81 cd	367.44 ± 11.77 ab	197.08 ± 6.59 d	284.48 ± 0.19 c	242.72 ± 5.82 cd	444.39 ± 54.83 a	260.87 ± 38.20 cd	358.56 ± 5.62 b	434.89 ± 35.11 ab	80.93 ± 8.47 e
67	2-Methylbutanal-M	67.66 ± 12.79 cd	81.20 ± 6.52 bc	90.42 ± 9.57 b	45.27 ± 1.54 ef	60.67 ± 6.97 de	32.05 ± 6.42 f	82.7 ± 22.36 bc	97.07 ± 1.30 b	81.82 ± 1.32 bc	282.46 ± 4.60 a
68	3-Methylbutanal-M	2325.54 ± 138.70 a	235.76 ± 6.40 f	1271.08 ± 183.12 c	390.72 ± 39.61 ef	681.00 ± 37.71 de	599.48 ± 185.74 de	1620.98 ± 19.17 b	1725.97 ± 35.02 b	744.23 ± 6.22 d	1216.56 ± 30.48 c
70	Oct-1-en-3-ol	133448.79 ± 108.23 a	9998.93 ± 123.24 d	11452.82 ± 419.99 b	10429.96 ± 138.46 cd	10916.61 ± 68.99 bc	10998.83 ± 527.14 bc	98.42 ± 22.00 e	114.65 ± 14.83 e	111.56 ± 17.34 e	123.19 ± 20.26 e

Each value is expressed as the mean ± standard deviation. Means with different lowercase letters within a row indicate significant differences (*p* < 0.05).

**Table 2:** Relative contents of volatile compounds identified from shiitake mushrooms treated with UVC-LED.

Group	Relative Contents (%)						
	Alcohols	Aldehydes	Ketones	Esters	Ethers	Others	Unidentified
C-CK	29.40 ± 1.97 a	44.75 ± 1.35 g	4.47 ± 0.36 e	8.04 ± 0.84 a	5.77 ± 0.57 d	2.85 ± 0.33 bc	4.72 ± 0.13 ef
S-CK	27.19 ± 1.00 b	57.16 ± 1.05 cd	4.35 ± 0.18 e	1.78 ± 0.21 f	0.24 ± 0.05 g	3.89 ± 0.12 a	5.40 ± 0.01 de
CF-30	29.13 ± 1.45 a	49.17 ± 1.32 f	7.06 ± 0.63 d	2.76 ± 0.20 e	3.32 ± 0.34 e	2.36 ± 0.17 d	6.19 ± 0.55 bc
SF-30	24.46 ± 0.39 c	58.00 ± 0.67 c	7.35 ± 0.22 cd	1.63 ± 0.01 f	0.33 ± 0.09 g	2.35 ± 0.06 d	5.88 ± 0.21 cd
CF-120	29.14 ± 1.14 a	51.92 ± 1.12 e	8.54 ± 0.23 ab	1.86 ± 0.08 f	0.69 ± 0.05 g	1.69 ± 0.05 e	6.16 ± 0.25 bcd
SF-120	25.01 ± 0.28 c	55.26 ± 1.58 d	8.60 ± 0.73 ab	1.84 ± 0.21 f	0.31 ± 0.01 g	2.28 ± 0.17 d	6.71 ± 0.67 b
CS-30	4.45 ± 0.57 e	58.29 ± 0.60 c	8.70 ± 0.52 a	4.94 ± 0.64 d	11.72 ± 0.62 b	2.77 ± 0.09 c	9.13 ± 0.56 a
CS-120	4.31 ± 0.53 e	56.85 ± 1.46 cd	7.90 ± 0.17 bc	5.98 ± 0.32 c	12.61 ± 0.32 a	3.11 ± 0.21 b	9.25 ± 0.37 a
SS-30	4.40 ± 0.64 e	61.92 ± 1.19 b	7.88 ± 0.51 bc	6.82 ± 0.61 b	10.32 ± 0.24 c	2.29 ± 0.27 d	6.38 ± 0.54 bc
SS-120	8.07 ± 0.51 d	79.17 ± 1.33 a	3.71 ± 0.23 e	2.32 ± 0.26 ef	1.41 ± 0.27 f	1.11 ± 0.24 f	4.21 ± 0.48 f

Each value is expressed as the mean ± standard deviation. Means with different lowercase letters within a column indicate significant differences (*p* < 0.05).

**Table 3:** Effects of UVC-LED treatment on vitamin D_2_ and ergosterol contents in shiitake mushrooms.

Groups	Vitamin D_2_ Content (μg/g)	Ergosterol Content (μg/g)
C-CK	0.00 ± 0.00 e	4275.25 ± 802.16 a
S-CK	0.00 ± 0.00 e	2144.41 ± 453.75 bc
CF-30	26.55 ± 1.30 e	2138.95 ± 75.33 bc
SF-30	29.25 ± 4.12 e	2295.13 ± 301.41 bc
CF-120	48.12 ± 7.00 e	2671.42 ± 292.59 b
SF-120	24.94 ± 12.92 e	2128.21 ± 254.84 bc
CS-30	267.85 ± 38.91 c	2595.18 ± 382.38 b
SS-30	112.60 ± 9.30 d	995.31 ± 80.81 cd
CS-120	668.79 ± 77.69 a	1603.58 ± 182.26 c
SS-120	399.45 ± 65.05 b	809.19 ± 123.30 d

Each value is expressed as the mean ± standard deviation. Means with different lowercase letters within a column indicate significant differences (*p* < 0.05).

## Data Availability

Data will be made available on request.

## Supplementary Materials

The following supporting information can be downloaded at: https://www.mdpi.com/article/10.3390/molecules29081872/s1, Figure S1: 3D-topographic plots for various parts of shiitake mushroom samples treated with UVC-LED. Figure S2: GC-IMS characterization of volatile components in fresh shiitake mushroom caps. Table S1: Volatile compounds in shiitake mushrooms identified by GC-IMS. Table S2: Peak volume of whole volatile compounds identified from shiitake mushroom with different treatments.

## References

[1] Luo D., Wu J., Ma Z., Tang P., Liao X., Lao F. (2021). Production of high sensory quality shiitake mushroom (*Lentinus edodes*) by pulsed air-impingement jet drying (AID) technique. Food Chem..

[2] Li B., Liu C., Fang D., Yuan B., Hu Q., Zhao L. (2019). Effect of boiling time on the contents of flavor and taste in *Lentinus edodes*. Flavour Fragr. J..

[3] Zhang Z., Lv G., Pan H., Wu Y., Fan L. (2009). Effects of different drying methods and extraction condition on antioxidant properties of shiitake (*Lentinus edodes*). Food Sci. Technol. Res..

[4] Li W., Chen W., Yang Y., Zhang J., Feng J., Yu H., Zhou S., Li X., Liu Y. (2017). Effects of culture substrates on taste component content and taste quality of *Lentinula edodes*. Int. J. Food Sci. Technol..

[5] Shi D., Zhou R., Feng X., Dong X., Gao H., Fan X., Yin C. (2020). Effects of low-dose γ-irradiation on the water state of fresh *Lentinula edodes*. LWT Food Sci. Technol..

[6] Hu D., Chen W., Li X., Yue T., Zhang Z., Feng Z., Li C., Bu X., Li Q., Hu C. (2020). Ultraviolet Irradiation increased the concentration of vitamin D2 and decreased the concentration of ergosterol in shiitakemushroom (*Lentinus edodes*) and oyster mushroom (*Pleurotus ostreatus*) powder in ethanol suspension. ACS Omega.

[7] Hu D., Yang X., Hu C., Feng Z., Chen W., Shi H. (2021). Comparison of ergosterol and vitamin D_2_ in mushrooms *Agaricus bisporus* and *Cordyceps militaris* using ultraviolet irradiation directly on dry powder or in ethanol suspension. ACS Omega.

[8] Hsu T.C., Teng Y.T., Yeh Y.W., Fan X.T., Chu K.H., Lin S.H., Yeh K.K., Lee P.T., Lin Y., Chen Z. (2021). Perspectives on UVC LED: Its progress and application. Photonics.

[9] Haley O.C., Pliakoni E.D., Rivard C., Nwadike L., Bhullar M. (2023). The attenuation of microbial reduction in blueberry fruit following UV-LED treatment. J. Food Prot..

[10] Keshavarzfathy M., Taghipour F. (2019). Computational modeling of ultraviolet light-emitting diode (UV-LED) reactor for water treatment. Water Res..

[11] Rouhani S., Taghipour F. (2022). Modeling of UV-LED photocatalytic reactors for the degradation of gaseous volatile organic compounds (VOCs) in indoor environments. J. Environ. Chem. Eng..

[12] Gerchman Y., Mamane H., Friedman N., Mandelboim M. (2020). UV-LED disinfection of coronavirus: Wavelength effect. J. Photochem. Photobiol. B Biol..

[13] Xiaokang W., Brunton N.P., Lyng J.G., Harrison S.M., Carpes S.T., Papoutsis K. (2020). Volatile and non-volatile compounds of shiitake mushrooms treated with pulsed light after twenty-four hour storage at different conditions. Food Biosci..

[14] Feng D., Wang J., Ji X., Min W., Yan W. (2021). HS-GC-IMS detection of volatile organic compounds in yak milk powder processed by different drying methods. LWT Food Sci. Technol..

[15] Ge S., Chen Y., Ding S., Zhou H., Jiang L., Yi Y., Deng F., Wang R. (2020). Changes in volatile flavor compounds of peppers during hot air drying process based on headspace-gas chromatography-ion mobility spectrometry (HS-GC-IMS). J. Sci. Food Agric..

[16] Guo Y., Chen D., Dong Y., Ju H., Wu C., Lin S. (2018). Characteristic volatiles fingerprints and changes of volatile compounds in fresh and dried *Tricholoma matsutake* Singer by HS-GC-IMS and HS-SPME-GC-MS. J. Chromatogr. B Anal. Technol. Biomed. Life Sci..

[17] Guo X., Schwab W., Ho C.T., Song C., Wan X. (2021). Characterization of the aroma profiles of oolong tea made from three tea cultivars by both GC-MS and GC-IMS. Food Chem..

[18] Yang Y., Qian M.C., Deng Y., Yuan H., Jiang Y. (2022). Insight into aroma dynamic changes during the whole manufacturing process of chestnut-like aroma green tea by combining GC-E-Nose, GC-IMS, and GC × GC-TOFMS. Food Chem..

[19] Xi L., Zhang J., Wu R., Wang T., Ding W. (2021). Characterization of the volatile compounds of Zhenba bacon at different process stages using GC-MS and GC-IMS. Foods.

[20] Gu S., Chen W., Wang Z., Wang J., Huo Y. (2020). Rapid detection of Aspergillus spp. infection levels on milled rice by headspace-gas chromatography ion-mobility spectrometry (HS-GC-IMS) and E-nose. LWT Food Sci. Technol..

[21] Tata A., Massaro A., Damiani T., Piro R., Dall’Asta C., Suman M. (2022). Detection of soft-refined oils in extra virgin olive oil using data fusion approaches for LC-MS, GC-IMS and FGC-Enose techniques: The winning synergy of GC-IMS and FGC-Enose. Food Control.

[22] Daulton E., Wicaksono A., Bechar J., Covington J.A., Hardwicke J. (2020). The detection of wound infection by ion mobility chemical analysis. Biosensors.

[23] Gasparri R., Capuano R., Guaglio A., Caminiti V., Canini F., Catini A., Sedda G., Paolesse R., Di Natale C., Spaggiari L. (2022). Volatolomic urinary profile analysis for diagnosis of the early stage of lung cancer. J. Breath Res..

[24] Li M., Yang R., Zhang H., Wang S., Chen D., Lin S. (2019). Development of a flavor fingerprint by HS-GC-IMS with PCA for volatile compounds of *Tricholoma matsutake* Singer. Food Chem..

[25] Hadar Y., Dosoretz C.G. (1991). Mushroom mycelium as a potential source of food. Trends Food Sci. Technol..

[26] Cho I.H., Kim S.Y., Choi H.K., Kim Y.S. (2006). Characterization of aroma-active compounds in raw and cooked pine-mushrooms (*Tricholoma matsutake* sing.). J. Agric. Food Chem..

[27] Hofmann T., Munch P., Schieberle P. (2000). Quantitative model studies on the formation of aroma-active aldehydes and acids by Strecker-type reactions. J. Agric. Food Chem..

[28] Cao J., Zou X., Deng L., Fan Y., Li H., Li J., Deng Z. (2014). Analysis of nonpolar lipophilic aldehydes/ketones in oxidized edible oils using HPLC-QqQ-MS for the evaluation of their parent fatty acids. Food Res. Int..

[29] Fan X., Jiao X., Liu J., Jia M., Blanchard C., Zhou Z. (2021). Characterizing the volatile compounds of different sorghum cultivars by both GC-MS and HS-GC-IMS. Food Res. Int..

[30] Song J., Shao Y., Yan Y., Li X., Peng J., Guo L. (2021). Characterization of volatile profiles of three colored quinoas based on GC-IMS and PCA. LWT Food Sci. Technol..

[31] Yang X., Zhu K., Guo H., Geng Y., Lv W., Wang S., Guo Y., Qin P., Ren G. (2021). Characterization of volatile compounds in differently coloured Chenopodium quinoa seeds before and after cooking by headspace-gas chromatography-ion mobility spectrometry. Food Chem..

[32] Hiraide M., Miyazaki Y., Shibata Y. (2004). The smell and odorous components of dried shiitake mushroom, *Lentinula edodes* I: Relationship between sensory evaluations and amounts of odorous components. J. Wood Sci..

[33] Hou H., Liu C., Lu X., Fang D., Hu Q., Zhang Y., Zhao L. (2021). Characterization of flavor frame in shiitake mushrooms (*Lentinula edodes*) detected by HS-GC-IMS coupled with electronic tongue and sensory analysis: Influence of drying techniques. LWT Food Sci. Technol..

[34] Fukami K., Ishiyama S., Yaguramaki H., Masuzawa T., Nabeta Y., Endo K. (2002). Identification of distinctive volatile compounds in fish sauce. J. Agric. Food Chem..

[35] Liu J., Xue J.L., Feng C.P. (2018). Effects of different drying methods on volatile flavor compounds in *Lentinus edodes*. Sci. Technol. Food Ind..

[36] Tian Y., Zhao Y., Huang J., Zeng H., Zheng B. (2016). Effects of different drying methods on the product quality and volatile compounds of whole shiitake mushrooms. Food Chem..

[37] Mau J.L., Chen P.R., Yang J.H. (1998). Ultraviolet irradiation increased vitamin D_2_ content in edible mushrooms. J. Agric. Food Chem..

[38] Pei F., Shi Y., Gao X., Wu F., Mariga A.M., Yang W., Zhao L., An X., Xin Z., Yang F. (2014). Changes in non-volatile taste components of button mushroom (*Agaricus bisporus*) during different stages of freeze drying and freeze drying combined with microwave vacuum drying. Food Chem..

[39] Zhang H., Huang D., Pu D., Zhang Y., Chen H., Sun B., Ren F. (2020). Multivariate relationships among sensory attributes and volatile components in commercial dry porcini mushrooms (*Boletus edulis*). Food Res. Int..

