# Supplementary material for: Analysis of Volatile Aroma Components in Different Parts of Shiitake Mushroom (Lentinus edodes) Treated with Ultraviolet C Light-Emitting Diodes Based on Gas Chromatography–Ion Mobility Spectroscopy
Source: Molecules. 2024 Apr 19;29(8):1872. doi: 10.3390/molecules29081872 (PMC11053434; doi:10.3390/molecules29081872)
Supplement: Supplementary file 1 [file molecules-29-01872-s001.zip › molecules-2948722-supplementary.pdf]

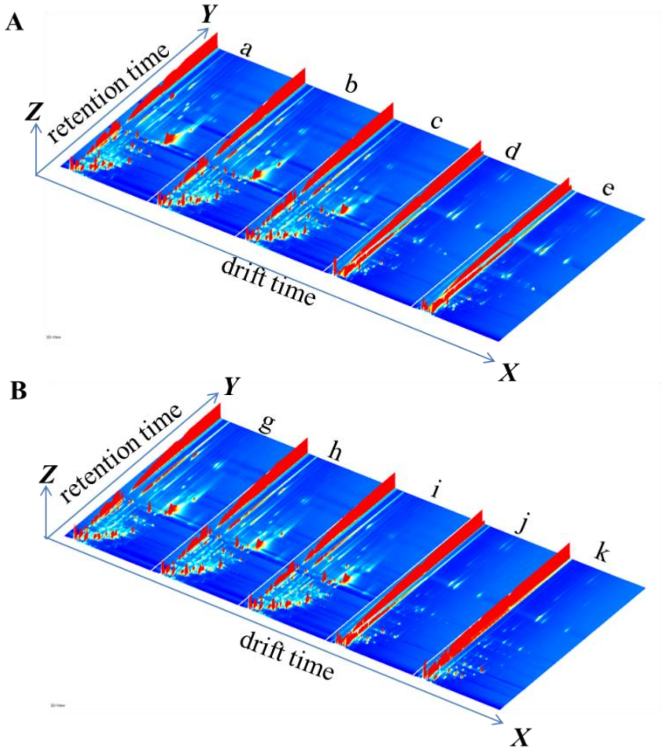

Figure S1. 3D-topographic plots for various parts of shiitake mushroom samples treated with UVC-LED.

A: Shiitake mushroom caps treatment samples. Lowercase letters a-e represent C-CK, CF-30, CF-120, CS-30, and CS-120, respectively; B: Shiitake mushroom stems treatment samples. Lowercase letters g-k represent C-CK, CF-30, CF-120, CS-30, and CS-120, respectively.

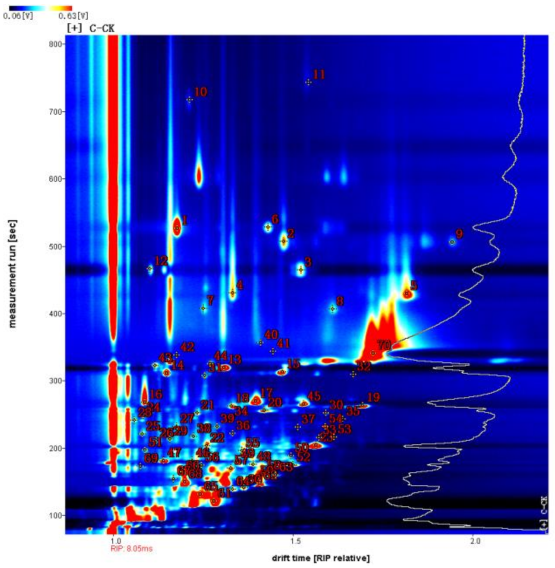

Figure S2. GC-IMS characterization of volatile components in fresh shiitake mushroom caps.

Table S1. Volatile compounds in shiitake mushrooms identified by GC-IMS.

No	Compounds	CAS#	Formula	M_W	RI	t_R (s)	Dt [a.u.]	Comment
Alcohols(13)								
10	(-)-Myrtenol	19894-97-4	$C_{10}H_{16}O$	152.2	1254.7	717.29	1.21511	
34	n-Hexanol-M	111-27-3	$C_6H_{14}O$	102.2	866.9	243.386	1.32884	Monomer
35	n-Hexanol-D	111-27-3	$C_6H_{14}O$	102.2	866.9	243.386	1.64073	Dimer
39	2-Methyl-1-pentanol-M	105-30-6	$C_6H_{14}O$	102.2	846.2	232.268	1.29126	Monomer
54	2-Methyl-1-pentanol-D	105-30-6	$C_6H_{14}O$	102.2	845.5	231.88	1.59225	Dimer
50	Pentan-1-ol	71-41-0	$C_5H_{12}O$	88.1	758	188.454	1.49677	
52	3-Methylbutan-1-ol-D	123-51-3	$C_5H_{12}O$	88.1	724	174.645	1.50244	Dimer
56	3-Methylbutan-1-ol-M	123-51-3	$C_5H_{12}O$	88.1	724.4	174.796	1.24763	Monomer
58	3-Pentanol-D	584-02-1	$C_5H_{12}O$	88.1	695.8	163.215	1.42007	Dimer
66	3-Pentanol-M	584-02-1	$C_5H_{12}O$	88.1	693.9	162.436	1.19391	Monomer
60	1-Propanethiol	107-03-9	C_3H_8S	76.2	621	142.063	1.36537	
63	Pentan-2-ol	6032-29-7	$C_5H_{12}O$	88.1	686.4	159.706	1.45384	
70	1-Octen-3-ol	3391-86-4	$C_8H_{16}O$	128.2	990.8	341.054	1.7244	
	Aldehydes(28)							
2	Nonanal-M	124-19-6	$C_9H_{18}O$	142.2	1108.3	506.763	1.47589	Monomer
9	Nonanal-D	124-19-6	$C_9H_{18}O$	142.2	1108	506.287	1.94515	Dimer
4	*E*-2-octenal-M	2548-87-0	$C_8H_{14}O$	126.2	1055.5	430.759	1.33448	Monomer
5	*E*-2-octenal-D	2548-87-0	$C_8H_{14}O$	126.2	1054.8	429.745	1.81812	Dimer
7	Benzene acetaldehyde	122-78-1	C_8H_8O	120.2	1039.3	407.441	1.25337	
11	Decanal	112-31-2	$C_{10}H_{20}O$	156.3	1272.9	743.387	1.54487	
14	Benzaldehyde-M	100-52-7	C_7H_6O	106.1	956.3	311.654	1.15082	Monomer
15	Benzaldehyde-D	100-52-7	C_7H_6O	106.1	957.7	312.861	1.47298	Dimer
16	3-Methylthiopropanal-M	3268-49-3	C_4H_8OS	104.2	904.8	267.688	1.09006	Monomer
17	3-Methylthiopropanal-D	3268-49-3	C_4H_8OS	104.2	907.1	269.621	1.39809	Dimer
18	Heptanal-M	111-71-7	$C_7H_{14}O$	114.2	898.9	262.616	1.33168	Monomer
19	Heptanal-D	111-71-7	$C_7H_{14}O$	114.2	899.7	263.34	1.69624	Dimer
22	Hexanal-M	66-25-1	$C_6H_{12}O$	100.2	792.2	203.362	1.26245	Monomer
23	Hexanal-D	66-25-1	$C_6H_{12}O$	100.2	795	204.844	1.56557	Dimer
25	Furfural-M	98-01-1	$C_5H_4O_2$	96.1	825.8	221.336	1.08459	Monomer
36	Furfural-D	98-01-1	$C_5H_4O_2$	96.1	827.5	222.262	1.3351	Dimer
27	*E*-2-hexenal-M	6728-26-3	$C_6H_{10}O$	98.1	845.8	232.083	1.17853	Monomer
37	*E*-2-hexenal-D	6728-26-3	$C_6H_{10}O$	98.1	844.8	231.527	1.51672	Dimer
31	*E*-hept-2-enal-M	18829-55-5	$C_7H_{12}O$	112.2	951.5	307.499	1.25744	Monomer
32	*E*-hept-2-enal-D	18829-55-5	$C_7H_{12}O$	112.2	953.9	309.538	1.66954	Dimer
40	Octanal	124-13-0	$C_8H_{16}O$	128.2	1003.8	356.427	1.41261	
51	3-Methyl-2-butenal-M	107-86-8	C_5H_8O	84.1	779.4	197.106	1.09028	Monomer
55	3-Methyl-2-butenal-D	107-86-8	C_5H_8O	84.1	777.3	196.254	1.35993	Dimer
59	2-Methylbutanal-D	96-17-3	$C_5H_{10}O$	86.1	668.7	154.927	1.40033	Dimer
67	2-Methylbutanal-M	96-17-3	$C_5H_{10}O$	86.1	666.2	154.245	1.17097	Monomer
61	Butanal	123-72-8	C_4H_8O	72.1	543.9	121.269	1.28346	

62	3-Methylbutanal-D	590-86-3	$C_5H_{10}O$	86.1	642	147.734	1.41015	Dimer
68	3-Methylbutanal-M	590-86-3	$C_5H_{10}O$	86.1	649	149.624	1.20155	Monomer
Ketones(7)								
26	4-Methyl-3-penten-2-one,	141-79-7	$C_6H_{10}O$	98.1	805.4	210.403	1.12217	
42	Methyl-5-hepten-2-one	110-93-0	$C_8H_{14}O$	126.2	987.6	338.34	1.17964	
44	1-Octen-3-one	4312-99-6	$C_8H_{14}O$	126.2	972.7	325.636	1.27176	
49	*E*-3-penten-2-one	3102-33-8	C_5H_8O	84.1	738.4	180.468	1.34647	
57	3-Hydroxybutan-2-one	513-86-0	$C_4H_8O_2$	88.1	712.9	170.137	1.32958	
65	2-Butanone	78-93-3	C_4H_8O	72.1	582.9	131.771	1.24524	
69	1-Penten-3-one	1629-58-9	C_5H_8O	84.1	720.3	173.148	1.07814	
Acids(2)								
21	Pentanoic acid	109-52-4	$C_5H_{10}O_2$	102.1	884.3	252.711	1.2356	
29	Butanoic acid	107-92-6	$C_4H_8O_2$	88.1	811.9	213.924	1.15974	
Ester(5)								
1	Tetrahydrofurfuryl acetate	637-64-9	$C_7H_{12}O_3$	144.2	1122.1	526.57	1.18018	
6	Hexyl propanoate	2445763	$C_9H_{18}O_2$	158.2	1123.5	528.591	1.43243	
38	Butyl acetate-M	123-86-4	$C_6H_{12}O_2$	116.2	818.2	217.259	1.22488	Monomer
53	Butyl acetate-D	123-86-4	$C_6H_{12}O_2$	116.2	815.9	216.074	1.61683	Dimer
64	Ethyl Acetate	141-78-6	$C_4H_8O_2$	88.1	609.3	138.912	1.3337	
Others(8)								
12	2-Acetylpyrrole	1072-83-9	C_6H_7NO	109.1	1080.3	466.389	1.10397	
13	Dimethyl trisulfide	3658-80-8	$C_2H_6S_3$	126.3	965.3	319.324	1.30946	
20	Styrene	100-42-5	C_8H_8	104.2	890.2	255.852	1.42212	
24	2,6-Dimethylpyridine	108-48-5	C_7H_9N	107.2	877	248.76	1.08584	
28	*P*-xylene	106-42-3	C_8H_{10}	106.2	862.8	241.163	1.06204	
43	3-Ethylpyridine	536-78-7	C_7H_9N	107.2	969	322.476	1.11873	
47	Dimethyl disulfide	624-92-0	$C_2H_6S_2$	94.2	740	181.134	1.14228	
48	Methanedithiol	6725-64-0	CH_4S_2	80.2	727.7	176.142	1.3909	
Unknown(7)								
3	Unknown-1	unidentified	*	0	1078.8	464.214	1.52426	
8	Unknown-2	unidentified	*	0	1038.6	406.428	1.61303	
30	Unknown-3	unidentified	*	0	883.9	252.466	1.59313	
33	Unknown-4	unidentified	*	0	815.4	215.777	1.57434	
41	Unknown-5	unidentified	*	0	993.7	343.551	1.44738	
45	Unknown-6	unidentified	*	0	902.1	265.349	1.53198	
46	Unknown-7	unidentified	*	0	740.8	181.466	1.22168	

M_W: The molecular weight of the identified compound. RI: The retention index was calculated using n-ketones C4-C9 as the external standard; t_R: The retention time in the capillary GC column; Dt: The drift time in the drift tube.

Table S2. Peak volume of whole volatile compounds identified from shiitake mushroom with different treatments

No.	Compound	Peak volume									
		C-CK	S-CK	CF-30	SF-30	CF-120	SF-120	CS-30	CS-120	SS-30	SS-120
1	Tetrahydrofurfuryl acetate	3445.46±268.46a	285.17±68.18cd	1018.88±97.13b	320.56±16.65cd	487.24±51.72c	346.95±19.49cd	76.27±8.75d	81.79±8.42d	77.14±14.31d	64.15±9.44d
2	Nonanal-M	1121.87±123.36bc	516.68±24.14d	560.05±18.70d	471.88±11.39d	580.09±8.82d	470.64±11.70d	1217.54±24.74b	990.63±18.69c	1165.60±35.60b	1406.95±72.82a
3	Unknown-1	845.46±16.37b	718.30±80.44c	1319.24±34.26a	557.04±13.04d	917.80±3.96b	461.31±88.52d	52.87±13.74e	61.01±13.44e	56.81±12.07e	52.49±11.36e
4	(E)-2-octenal-M	1168.89±82.60d	1439.12±51.14abc	1522.94±154.57ab	1613.89±54.92a	1370.27±39.28bc	1267.03±47.78cd	96.14±8.12e	91.55±3.36e	159.89±8.52e	278.88±16.30e
5	(E)-2-octenal-D	1697.18±164.73bc	2554.85±171.59a	2135.44±388.78ab	2071.31±111.03b	1173.13±88.40d	1411.05±101.22cd	65.86±18.53e	54.98±8.05e	63.37±13.20e	59.04±9.26e
6	Hexyl propanoate	580.36±114.20a	61.77±2.39b	72.88±11.93b	50.45±3.27b	53.24±7.31b	41.88±0.47b	74.43±11.04b	85.02±5.34b	55.05±5.50b	48.57±9.71b
7	benzene Acetaldehyde	379.25±93.68cd	481.88±48.34cd	586.78±125.12c	1139.66±59.77b	1379.87±170.48a	1607.23±50.99a	244.10±7.28d	252.32±3.16d	251.68±4.45d	287.97±6.43d
8	Unknown-2	575.22±117.71d	953.49±60.18c	864.83±122.62c	1199.14±56.24b	1051.40±80.77bc	1518.86±105.29a	49.72±11.85e	57.29±14.46e	50.22±11.27e	57.64±14.01e
9	Nonanal-D	272.84±52.40e	88.75±6.61f	87.14±5.61f	81.51±2.46f	92.97±7.73f	78.77±6.15f	1081.37±87.72b	1241.88±61.88a	714.00±18.51c	414.17±33.32d
10	(-)-Myrtenol	56.02±5.86b	18.71±2.44de	91.30±1.60a	36.45±6.49c	52.69±6.92b	29.61±1.46cd	8.83±2.43e	9.89±2.17e	10.39±3.01e	9.97±3.08e
11	Decanal	95.36±11.46d	83.20±3.73de	75.37±2.82e	65.86±4.10e	72.25±1.06e	66.80±5.30e	247.95±8.09a	193.45±7.85b	191.87±3.24b	144.32±4.29c
12	2-Acetylpyrrole	341.68±7.76a	81.52±5.98d	184.04±19.13b	74.71±2.24de	114.32±11.76c	70.79±1.10def	44.34±4.27fg	48.89±7.99efg	38.49±9.83g	30.38±7.10g
13	Dimethyl trisulfide	1939.56±210.48a	75.28±14.95cd	1317.78±179.27b	136.24±29.19cd	320.56±8.50c	110.19±11.91cd	19.67±3.54e	22.55±3.76e	21.96±4.91e	23.99±3.43e
14	Benzaldehyde-M	1181.71±74.24c	1705.72±21.93a	1519.95±77.50b	1551.58±58.27b	1535.48±64.81b	1127.15±6.67c	617.44±34.38e	885.01±3.00d	535.16±5.48e	375.28±15.75f

15	Benzaldehyde-D	1309.61±201.87e	5967.98±89.55b	2769.03±267.23d	5911.48±252.61b	3537.58±370.12c	7826.78±305.84a	63.54±11.97f	65.07±8.52f	86.55±10.32f	97.19±12.23f
16	3-methylthiopropanal-M	2060.40±54.81a	1862.86±85.26bc	1811.65±33.68cd	1971.21±66.82ab	1701.54±19.39d	1504.86±14.04e	185.45±16.36f	201.80±11.49f	142.08±6.67f	88.64±8.94f
17	3-methylthiopropanal-D	2391.70±204.38ab	1389.96±172.01d	1878.13±134.58c	2624.71±272.39a	2172.21±44.37bc	1867.35±194.27c	90.70±8.32e	118.42±4.88e	88.21±4.90e	69.85±12.03e
18	Heptanal-M	538.02±25.97bc	497.75±6.49c	584.33±31.76ab	569.29±12.55b	629.38±18.00a	541.30±17.20bc	83.13±2.44e	82.05±3.74e	89.25±2.92e	255.95±25.39d
19	Heptanal-D	690.93±76.69ab	466.91±27.29b	625.21±158.82ab	745.46±30.19a	718.12±76.75ab	870.98±170.07a	41.27±8.12c	66.50±4.50c	33.41±1.56c	70.78±6.96c
20	Styrene	420.30±22.33b	615.44±6.96a	277.23±7.73d	370.65±10.91c	255.50±11.94d	432.15±5.56b	23.36±3.87e	29.38±2.59e	24.78±4.86e	19.34±3.89e
21	pentanoic acid	204.76±6.90b	288.42±2.33a	233.89±10.74b	228.16±6.69b	160.93±1.18c	207.36±29.44b	14.24±2.90d	14.11±3.46d	13.53±2.37d	24.58±4.61d
22	Hexanal-M	618.48±25.93c	737.40±4.53b	709.01±17.57b	702.42±5.88b	698.98±25.77b	593.26±24.45c	228.17±6.00e	190.49±4.53e	335.94±0.84d	978.51±30.60a
23	Hexanal-D	2452.06±73.18e	2889.71±74.46d	3268.57±144.72c	3971.58±24.78ab	3709.24±110.91b	4103.04±178.64a	366.11±7.92h	315.11±8.66h	729.43±30.78g	2032.37±86.84f
24	2,6-Dimethylpyridine	64.64±5.38e	463.89±7.79a	138.36±2.93d	308.28±8.41b	74.94±7.02e	242.34±4.52c	16.54±0.57f	15.58±0.87f	16.35±0.94f	7.63±1.86f
25	Furfural-M	100.72±13.37c	120.93±14.26c	101.73±19.38c	261.37±31.57b	88.32±6.84c	321.28±27.19a	24.37±0.39d	21.62±0.29d	23.06±1.10d	19.52±1.70d
26	4-Methyl-3-penten-2-one	61.40±6.00d	24.26±0.97e	18.09±1.79ef	11.07±1.46f	13.40±1.18f	7.83±0.45f	379.84±2.46b	397.15±2.11a	308.27±5.17c	52.42±6.00d
27	(E)-2-hexenal-M	121.29±11.45d	152.87±7.28d	189.95±8.20c	249.37±1.48ab	229.84±9.64b	262.47±25.82a	35.91±1.64e	41.70±2.49e	40.69±1.39e	128.48±7.16d
28	*p*-Xylene	57.92±5.80b	70.89±3.02a	43.00±0.93c	49.43±0.91c	42.25±2.67c	61.89±1.80b	14.94±1.51de	10.04±0.80e	18.44±1.70d	16.39±1.48de
29	Butanoic acid	34.27±8.30cd	38.68±7.69cd	47.61±3.26bc	53.58±1.87b	40.60±2.31bcd	45.68±6.32bc	46.42±2.98bc	69.20±1.41a	30.17±0.82d	7.32±1.04e
30	Unknown-3	52.04±3.80e	172.83±14.85b	97.40±8.16d	144.73±1.52c	90.22±9.99d	216.38±11.58a	13.80±2.66f	12.83±2.09f	13.87±3.02f	13.99±4.53f
31	(E)-hept-2-enal-M	142.41±13.90de	178.22±11.24c	319.63±17.83a	245.20±5.30b	351.39±14.15a	273.04±15.24b	107.15±2.01f	113.12±1.94ef	163.73±1.72cd	325.56±8.09a
3	(E)-hept-2-e	46.13±4.	103.62±	263.01±	323.68±3	489.49±	472.03±7	19.21±	24.69±	30.92±	68.96±

2	nal-D	42c	7.19c	28.58b	4.64b	91.53a	3.36a	3.75c	1.98c	4.34c	4.97c
33	Unknown-4	67.32±5.64fg	138.87±5.27e	196.53±20.60d	435.98±14.24b	377.23±38.56c	745.67±23.32a	94.89±5.66efg	100.89±3.58efg	111.92±2.86ef	52.78±1.13g
34	n-Hexanol-M	59.00±5.57c	157.05±1.42c	383.12±75.98b	368.33±16.53b	651.51±21.53a	489.45±98.70b	43.48±2.19c	49.63±1.82c	43.87±4.58c	48.40±5.66c
35	n-Hexanol-D	10.04±1.06c	15.72±1.20c	81.98±31.32bc	95.12±5.49b	264.23±14.76a	242.05±61.75a	7.73±1.05c	7.76±1.32c	8.99±1.68c	7.50±1.64c
36	Furfural-D	76.61±6.25de	181.83±4.04bc	116.26±4.81cd	249.09±48.42b	112.30±6.47cd	445.62±57.60a	40.67±4.75de	42.04±3.86de	39.03±4.67de	27.73±5.15e
37	(E)-2-hexenal-D	27.52±3.72ef	53.66±2.95de	83.78±10.10cd	110.00±10.99bc	127.76±17.71b	226.93±27.99a	14.89±2.42f	11.48±1.96f	14.08±1.89f	19.49±2.94ef
38	Butyl acetate-M	119.52±7.23d	129.95±1.83d	170.68±8.33c	210.92±4.21b	212.22±8.27b	242.73±2.18a	33.71±2.32f	31.78±1.99f	35.67±1.46f	67.77±3.22e
39	2-Methyl-1-pentanol-M	79.71±7.90cd	59.73±4.66de	99.18±16.15bc	129.03±6.74b	124.78±11.50b	171.66±23.62a	41.14±2.08f	41.99±1.20f	44.32±1.48f	38.70±2.47f
40	Octanal	60.30±1.75d	49.91±0.87d	51.95±9.19d	67.64±6.67d	59.32±4.80d	57.50±16.00d	163.80±1.95b	117.29±0.83c	140.04±3.61bc	272.04±20.00a
41	Unknown-5	34.38±3.57c	33.39±3.08c	37.54±3.34c	37.84±2.32c	37.33±2.24c	49.42±2.15c	549.68±35.74a	578.35±12.39a	271.54±12.76b	33.75±6.02c
42	Methyl-5-hepten-2-one	19.66±1.62e	14.80±0.72e	17.24±2.10e	21.36±3.22e	26.78±1.98e	21.15±1.82e	168.53±6.90a	101.81±1.52d	132.11±9.67b	114.82±3.52c
43	3-Ethylpyridine	80.01±15.51a	52.74±10.14bc	39.83±18.10cde	22.96±2.63def	25.40±4.57cdef	18.75±1.22ef	78.03±4.41ab	96.22±4.60a	48.39±3.56cd	7.67±1.13f
44	1-Octen-3-one	112.06±5.94a	74.90±2.61d	68.60±7.96d	82.46±3.19cd	103.42±9.89ab	93.74±6.26bc	26.39±1.87e	23.24±2.78e	31.17±0.93e	75.49±1.79d
45	Unknown-6	900.22±72.05a	569.44±44.23b	732.91±146.97ab	907.31±33.19a	824.09±69.82ab	989.54±188.22a	19.72±4.98f	21.07±4.93f	21.00±5.00f	23.39±4.19f
46	Unknown-7	94.40±0.79d	135.68±2.97b	91.13±4.40d	117.84±3.45bc	101.47±8.39cd	110.07±9.67cd	48.45±3.69e	40.16±1.30e	45.05±2.55e	252.17±14.04a
47	Dimethyl disulfide	1212.54±88.71a	43.57±1.05e	480.13±29.66d	52.14±3.87e	60.25±6.07e	77.64±6.30e	1039.04±12.94b	1165.34±4.57a	898.34±8.86c	135.80±11.10e
48	Methanedithiol	339.27±48.80ab	350.30±10.90a	314.07±51.53ab	250.62±7.17bc	214.59±9.45c	311.56±51.66ab	13.62±1.12d	10.05±2.41d	15.22±2.33d	15.36±3.20d
49	(E)-3-penten-2-one	263.63±28.92cd	140.55±6.94d	322.13±74.48cd	616.49±23.37b	542.88±56.19bc	995.39±269.99a	95.94±11.10d	119.53±1.54d	91.80±0.82d	61.96±2.58d
50	Pentan-1-ol	170.56±13.90e	525.63±10.55c	657.20±63.22b	424.68±12.36d	840.45±49.90a	540.40±8.00c	18.54±4.42f	19.06±4.86f	18.69±4.85f	23.56±5.97f
5	3-Methyl-2-	60.74±1.	44.09±0.	47.29±7.	60.47±0.	47.13±3.	62.49±9.	29.54±	28.58±	30.25±	17.65±

1	butenal-M	88ab	62c	97bc	98ab	31bc	68a	1.49d	1.78d	2.04d	1.55d
52	3-Methylbutan-1-ol-D	391.74±79.46d	591.83±12.98c	916.28±101.16b	663.29±2.42c	1107.61±73.74a	702.73±26.14c	20.98±3.89e	15.13±1.99e	20.32±1.92e	17.93±1.33e
53	Butyl acetate-D	12.01±0.92d	52.89±2.90b	34.65±2.17c	77.43±10.01a	33.11±4.42c	38.30±1.98c	5.22±1.04d	5.64±1.87d	7.11±1.12d	6.87±2.67d
54	2-Methyl-1-pentanol-D	18.42±1.13e	21.78±2.12de	36.38±4.93cd	40.53±4.68bc	52.44±7.33b	75.20±11.49a	20.08±2.67e	19.78±1.06e	20.37±2.04e	13.60±1.00e
55	3-Methyl-2-butenal-D	81.43±5.08ab	52.63±2.15b	75.13±16.42ab	76.17±1.85ab	56.85±6.54b	102.57±32.82a	7.43±0.27c	8.79±0.86c	9.96±1.82c	15.85±3.75c
56	3-Methylbutan-1-ol-M	53.20±4.10c	88.17±6.12b	139.59±16.45a	104.54±0.16b	132.76±7.38a	90.52±20.47b	27.33±1.88cd	19.89±1.88d	30.52±3.07cd	35.52±2.66cd
57	3-Hydroxybutan-2-one	382.43±79.62a	261.94±11.26b	218.87±63.81bc	140.31±3.23c	132.56±4.93c	163.58±47.76bc	16.36±2.05d	16.47±0.98d	12.74±1.40d	11.03±1.16d
58	3-Pentanol-D	1047.13±28.56c	1437.64±56.04a	1062.02±84.19c	1236.07±20.39b	1053.83±55.02c	1119.73±10.82c	17.85±2.23e	16.12±1.14e	15.72±2.09e	213.51±20.96d
59	2-Methylbutanal-D	1720.30±67.75b	2178.78±48.23a	1668.37±110.67b	1871.52±29.66b	1784.22±87.06b	1744.87±123.04b	60.93±7.14d	51.11±3.11d	193.35±6.47d	737.99±61.36c
60	1-Propanethiol	304.35±7.10bc	308.96±15.62bc	368.94±32.98bc	263.74±9.76c	506.33±11.65a	408.44±112.13ab	14.19±1.34d	11.57±2.45d	15.05±1.47d	10.89±2.76d
61	Butanal	2682.47±518.61b	2409.04±178.95b	2081.47±241.56b	4111.36±53.03a	3568.64±166.36a	4146.59±247.11a	25.59±5.47c	21.05±4.91c	29.04±6.09c	67.72±12.81c
62	3-Methylbutanal-D	898.04±95.95d	2306.82±17.47a	1336.14±128.46c	1994.18±62.13b	1594.98±59.03c	1501.35±211.00c	36.41±3.38f	27.54±2.98f	139.93±3.26f	552.40±19.94e
63	Pentan-2-ol	68.58±5.85c	214.00±14.11a	66.41±8.09c	132.10±4.48b	72.56±7.72c	159.22±37.33b	4.93±1.54d	4.42±0.84d	5.35±0.80d	6.10±0.77d
64	Ethyl Acetate	235.71±12.81cd	367.44±11.77ab	197.08±6.59d	284.48±0.19c	242.72±5.82cd	444.39±54.83a	260.87±38.20cd	358.56±5.62b	434.89±35.11ab	80.93±8.47e
65	2-Butanone	1536.93±57.85c	1608.96±26.59c	3101.44±167.82b	3303.74±59.80b	3755.98±63.39a	3877.80±104.91a	80.32±2.24d	71.38±3.00d	104.49±4.52d	96.09±6.17d
66	3-Pentanol-M	325.80±6.50b	262.62±11.35c	322.69±6.17b	226.00±1.17d	267.70±6.10c	191.43±15.30e	81.76±3.21f	76.89±4.52f	48.93±0.87f	381.82±26.90a
67	2-Methylbutanal-M	67.66±12.79cd	81.20±6.52bc	90.42±9.57b	45.27±1.54ef	60.67±6.97de	32.05±6.42f	82.7±22.36bc	97.07±1.30b	81.82±1.32bc	282.46±4.60a
68	3-Methylbutanal-M	2325.54±138.70a	235.76±6.40f	1271.08±183.12c	390.72±39.61ef	681.00±37.71de	599.48±185.74de	1620.98±19.17b	1725.97±35.02b	744.23±6.22d	1216.56±30.48c
6	1-Penten-3-	46.99±2.	65.36±1.	67.87±6.	76.19±4.	126.86±	90.09±11	18.51±	13.70±	20.66±	13.29±

9	one	45d	36c	56c	06c	3.25a	.82b	0.63e	0.31e	0.30e	0.57e
70	Oct-1-en-3-ol	133448.79±108.23a	9998.93±123.24d	11452.82±419.99b	10429.96±138.46cd	10916.61±68.99bc	10998.83±527.14bc	98.42±22.00e	114.65±14.83e	111.56±17.34e	123.19±20.26e

Each value is expressed as the mean ± standard deviation. Means with different lowercase letters within a row indicate significant differences (p<0.05).
